# Supplementary material for: GPC2-CAR T cells have potent preclinical activity against orthotopic medulloblastoma xenografts
Source: Mol Ther Oncol. 2025 Sep 30;33(4):201067. doi: 10.1016/j.omton.2025.201067 (PMC12554907; doi:10.1016/j.omton.2025.201067)
Supplement: Document S1. Figures S1–S11 and Tables S1–S3 [file mmc1.pdf]

OMTON, Volume *33*

Supplemental information

GPC2-CAR T cells have potent preclinical activity against orthotopic medulloblastoma xenografts

Reona Okada, Mia Fanuzzi, Constanza Rodriguez, Jangsuk Oh, Adhithi Sreenivasan, Hannah G. Stack, Mariela Puebla, Ira Phadke, Allison P. Cole, Shanshan Bradford, Michael C. Kelly, Haiyan Lei, Mitchell Ho, Jennifer A. Cotter, Carol J. Thiele, Xiyuan Zhang, Anandani Nellan, and Rosa Nguyen

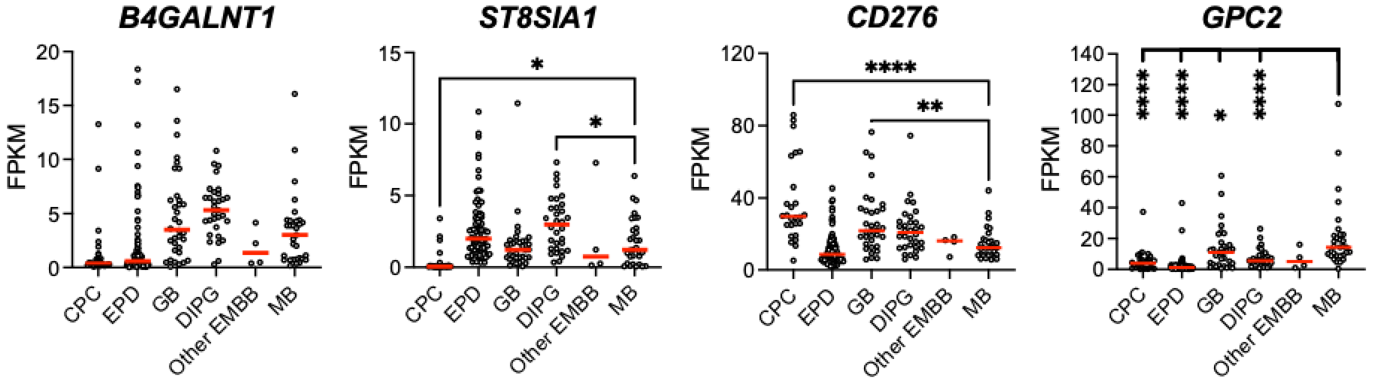

Figure S1: Expression of *B4GLANT1* and *ST8SIA1* (enzymes responsible for GD2 synthesis), *CD276*, and *GPC2* in various primary tumors and derived xenografts and cell lines from patients with choroid plexus carcinoma (CPC), ependymoma (EPD), glioblastoma (GB), diffuse intrinsic pontine glioma (DIPG), other embryonal brain tumors (EMBB), and medulloblastoma (MB) from primary patient samples obtained through the St. Jude Pediatric Cancer Knowledge Base portal.

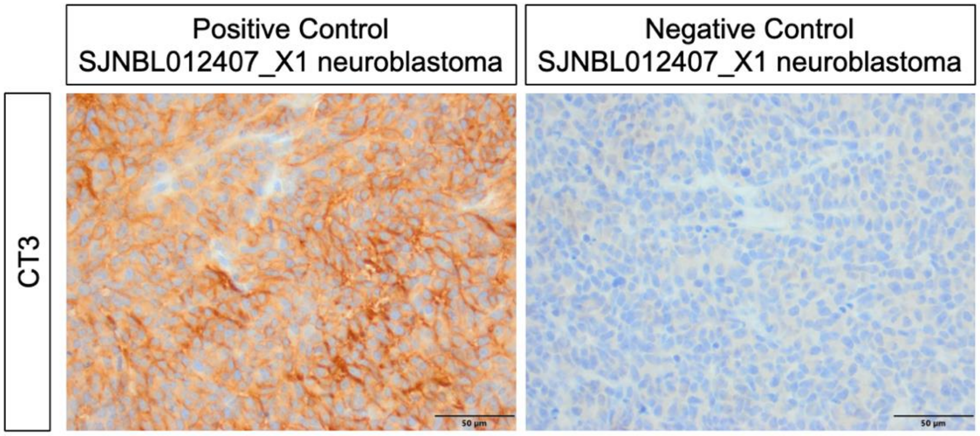

Figure S2: The positive control tissue consisted of SJNBL012407_X1 neuroblastoma cells, which demonstrated diffuse and strong CT3 positivity with a cytoplasmic to membranous staining pattern (CT3, 1:1000, BioX BE0402, mouse IgG1, using the M.O.M. ImmPRESS Kit and Bond Polymer Refine Detection Kit). The negative control was performed on the same tissue by substituting the primary antibody with a nonclonal, isotype-matched mouse antibody.

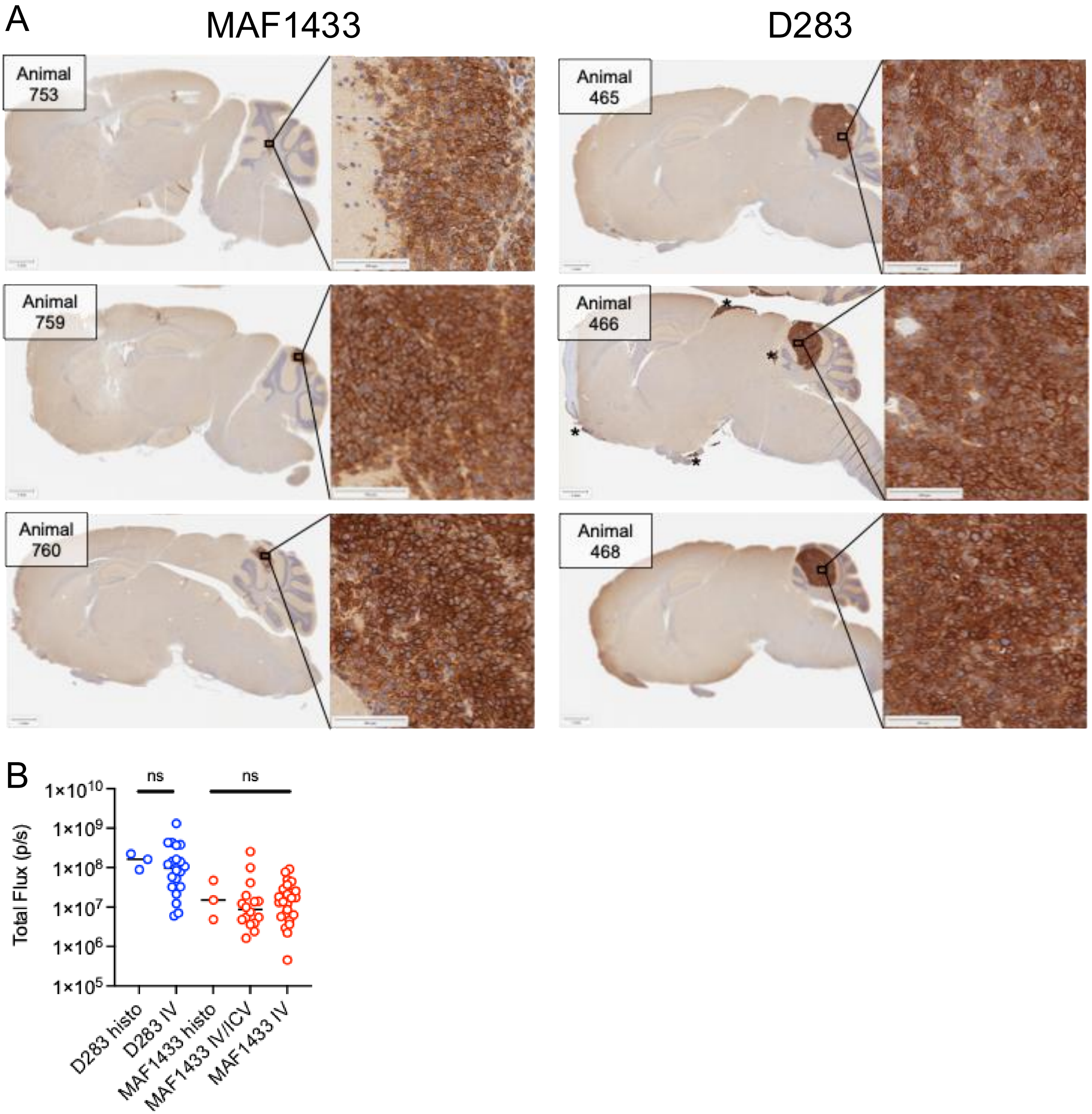

Figure S3: (A) *In vivo* GPC2 expression in MAF1433 and D283. Mice were injected with MAF1433 and D283 cells, and tumors were harvested at the time point corresponding to when CAR T-cell treatment would have been administered. Immunohistochemistry performed on these tissues revealed homogeneous and strong GPC2 expression in both models. **(B)** Animals shown in (A) were also subjected to IVIS bioluminescence imaging (BLI). The average BLI signal was compared to the baseline signal of mice treated in this study. No significant difference in average signal was observed, indicating comparable tumor burden across studies.

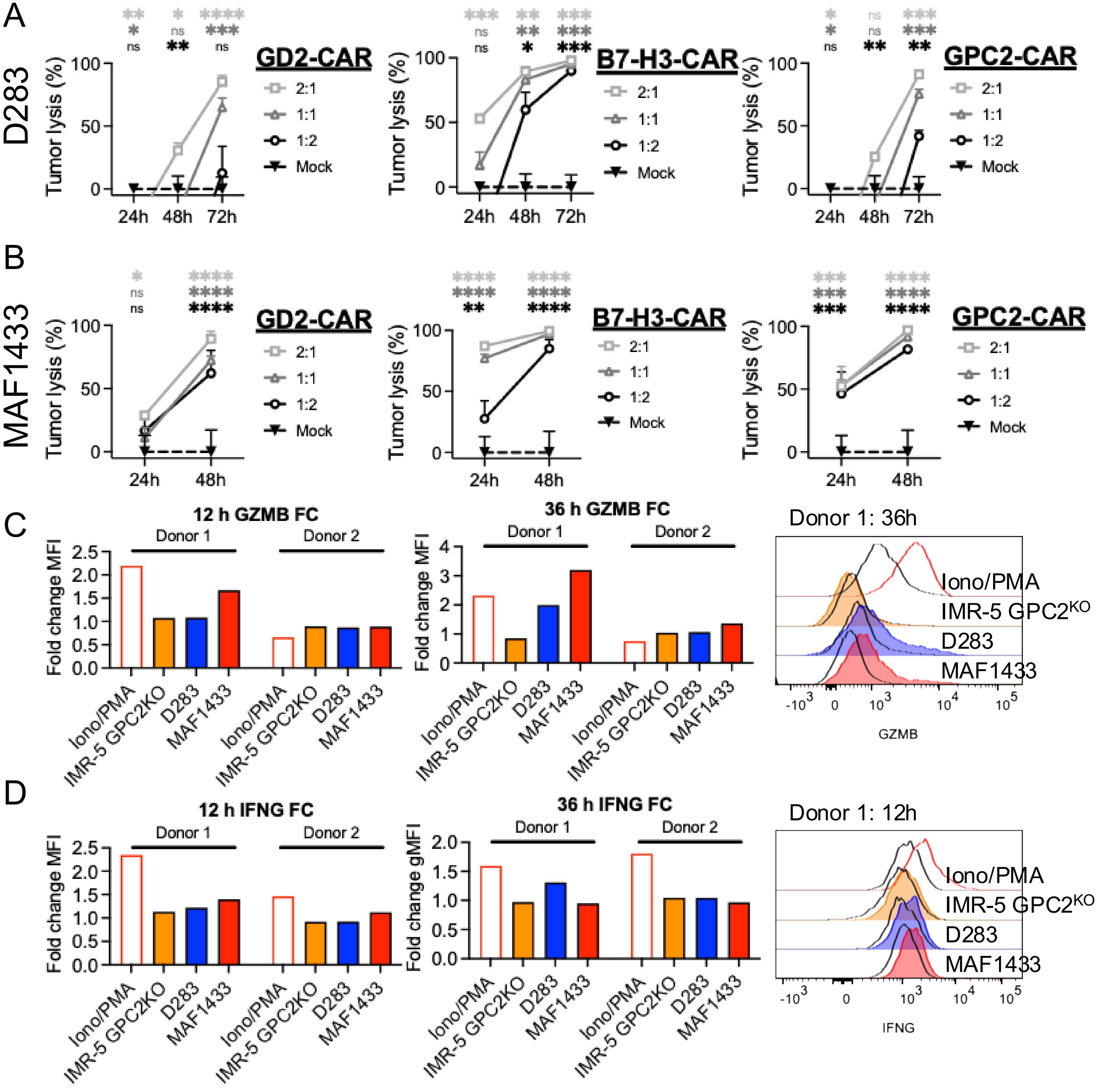

Figure S4: *In vitro* CAR T-cell killing against **(A)** D283 and **(B)** MAF1433 (second replicate with independent donor). Intracellular **(C)** granzyme B and **(D)** interferon gamma levels of GPC2-CAR T-cells compared to mock control T-cells are shown as fold change at 12 and 36 hours post-co-culture. A representative flow plot is shown for each cytokine. The black curve is the isotype control for each condition.

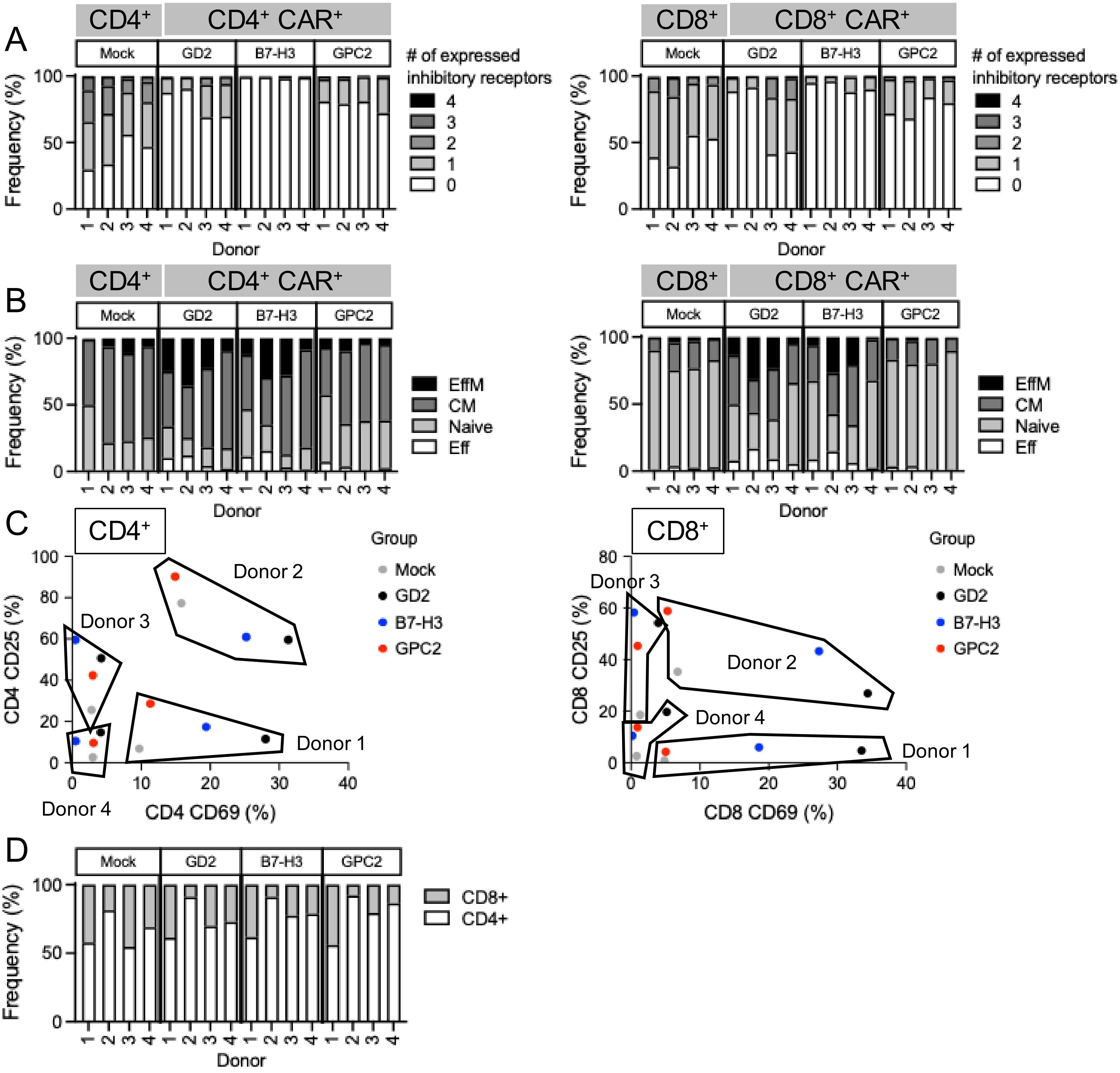

Figure S5: Phenotype analysis of different T-cell products after production. **(A)** The fraction of cells with single, double, triple, or quadruple positivity for LAG3, TIM3, CD39, and PD-1 is plotted for four independent donors and shown for CD4 and CD8 T-cells. **(B)** Fractions of $CD62L^-$ $CD45RA^-$ effector memory (EffM), $CD62L^+$ $CD45RA^-$ central memory (CM), $CD62L^+$ $CD45RA^+$ naïve, and $CD62L^-$ $CD45RA^+$ effector T-cells (Eff) across the four donors from (A). **(C)** CD25 and CD69 positivity across CARs and donors is shown for CD4 and CD8 T-cells separately. **(D)** Frequency of CD8- and CD4-positive T-cells by CAR group.

A **D283 rechallenge assay**

Tukey's multiple comparisons test	Mean Diff.	95.00% CI of diff.	Summary	Adj. P Value
Mock vs. B7-H3-CAR	34.17	28.15 to 40.20	****	<0.0001
Mock vs. GD2-CAR	28.36	22.91 to 33.81	****	<0.0001
Mock vs. GPC2-CAR	-11.36	-13.79 to -8.929	****	<0.0001
B7-H3-CAR vs. GD2-CAR	-7.330	-8.056 to -6.604	****	<0.0001
B7-H3-CAR vs. GPC2-CAR	-45.53	-53.89 to -37.18	****	<0.0001
GD2-CAR vs. GPC2-CAR	-39.72	-47.45 to -31.99	****	<0.0001

B **MAF1433 rechallenge assay**

Tukey's multiple comparisons test	Mean Diff.	95.00% CI of diff.	Summary	Adj. P Value
Mock vs. B7H3 CAR	4.714	3.991 to 5.438	****	<0.0001
Mock vs. GD2 CAR	4.572	3.773 to 5.370	****	<0.0001
Mock vs. GPC2 CAR	2.797	2.343 to 3.250	****	<0.0001
B7H3 CAR vs. GD2 CAR	-0.1426	-0.2402 to -0.04505	**	0.0014
B7H3 CAR vs. GPC2 CAR	-1.917	-2.195 to -1.640	****	<0.0001
GD2 CAR vs. GPC2 CAR	-1.775	-2.129 to -1.420	****	<0.0001

C

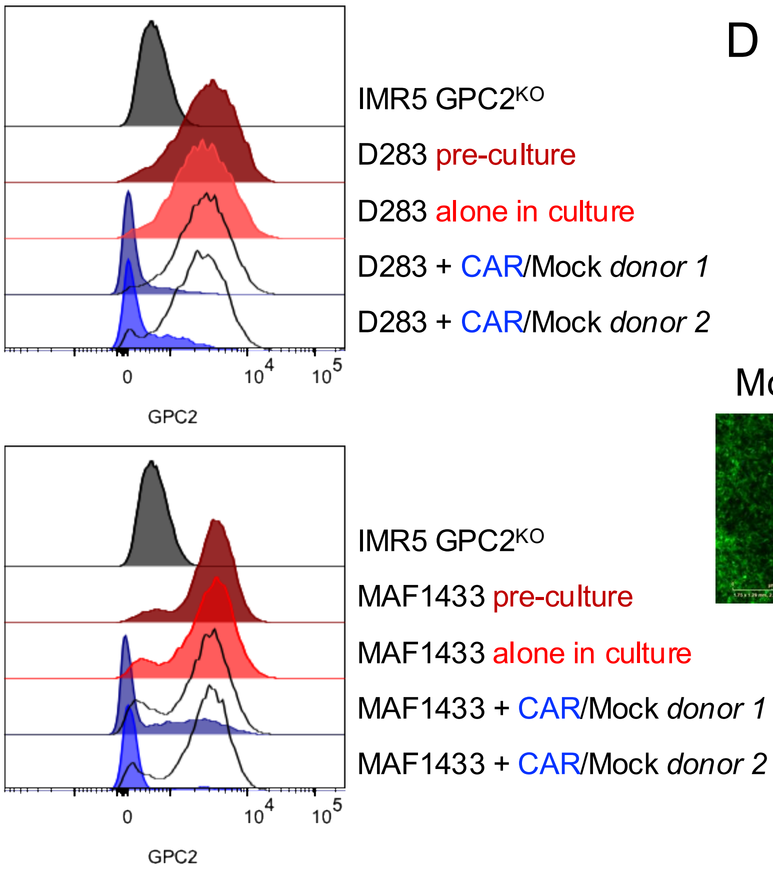

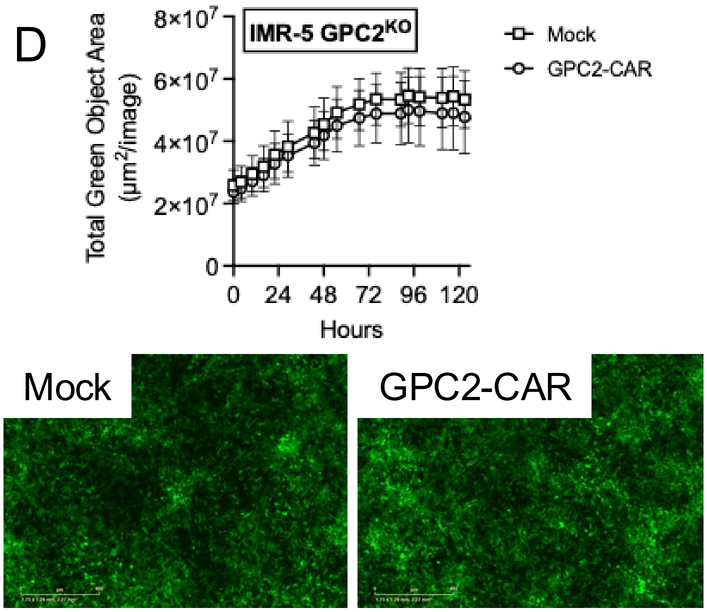

Figure S6: Tukey's multiple comparisons test of each treatment group for the **(A)** D283 and **(B)** MAF1433 tumor rechallenge assay. These statistics correspond to results shown in main Fig. 2E-F. **(C)** GPC2 expression in surviving D283 and MAF1433 cell clones after co-culture with Mock or GPC2-CAR T-cells. These cells downregulate GPC2 under CAR-mediated selection pressure. **(D)** GPC2-CAR T-cell killing against the GPC2 knockout line IMR-5 GPC2^{KO}. IMR-5 GPC2^{KO} cells are resistant to GPC2-CAR cytotoxicity. Using Incucyte live-cell imaging, tumor growth curves are similar for mock T-cell-treated or GPC2-CAR-treated conditions. Representative fluorescence images of a co-culture well are shown at the bottom panel, where green signals represent tumor cells.

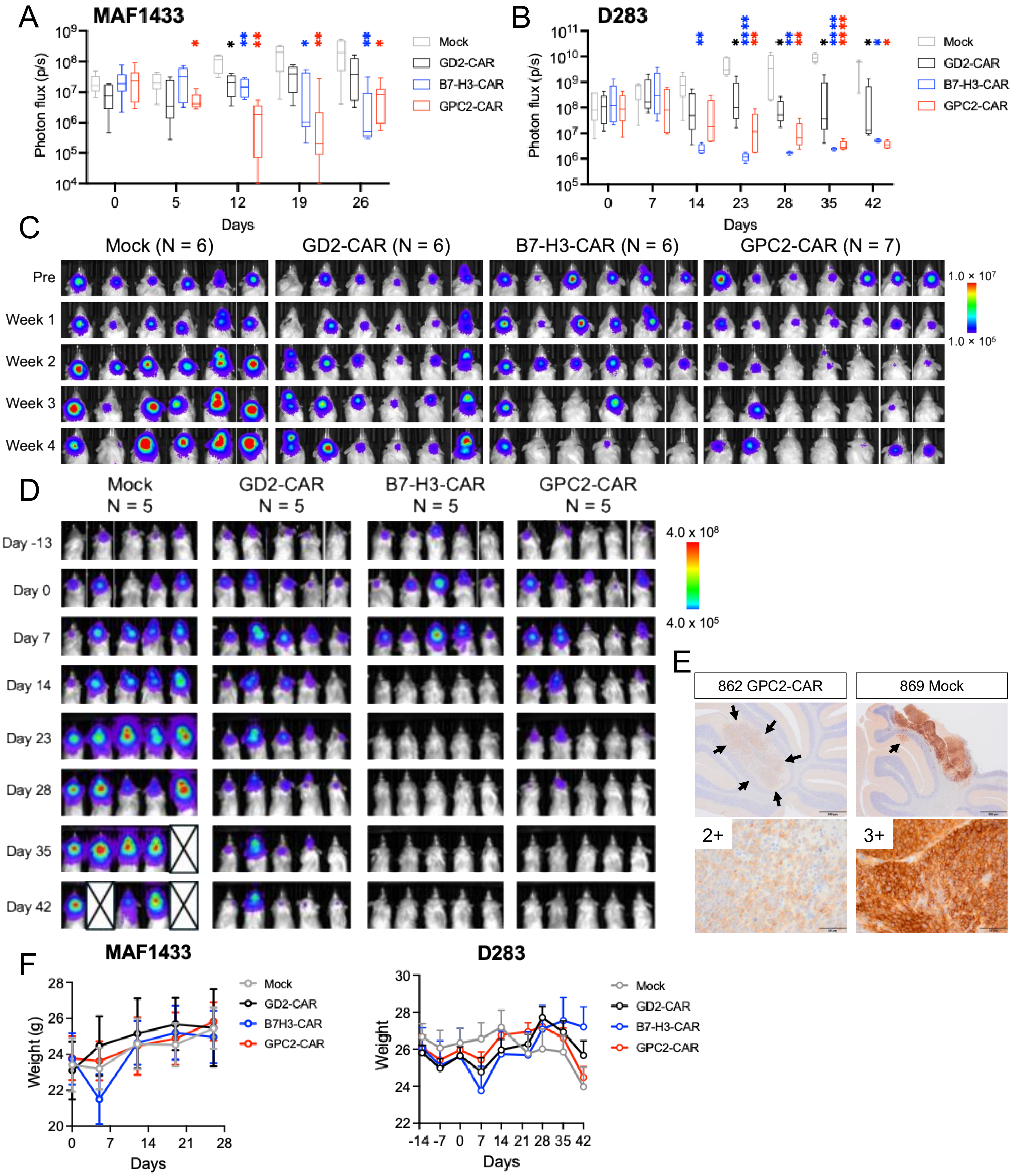

Figure S7: Bar graph plot of the BLI signal of **(A)** MAF1433 and **(B)** D283 tumors. Two-way ANOVA with multiple comparisons; * $P < 0.05$; ** $P < 0.01$; **** $P < 0.0001$. Corresponding IVIS bioluminescence images of **(C)** MAF1433 and **(D)** D283-bearing mice during CAR T-cell treatment. **(E)** Histological review of MAF1433 mice treated with GPC2-CAR and IVIS signals above background demonstrates tumor cells in the posterior fossa with marked downregulation of GPC2 compared to mock-treated animals. **(F)** Longitudinal body weight measurements of study mice.

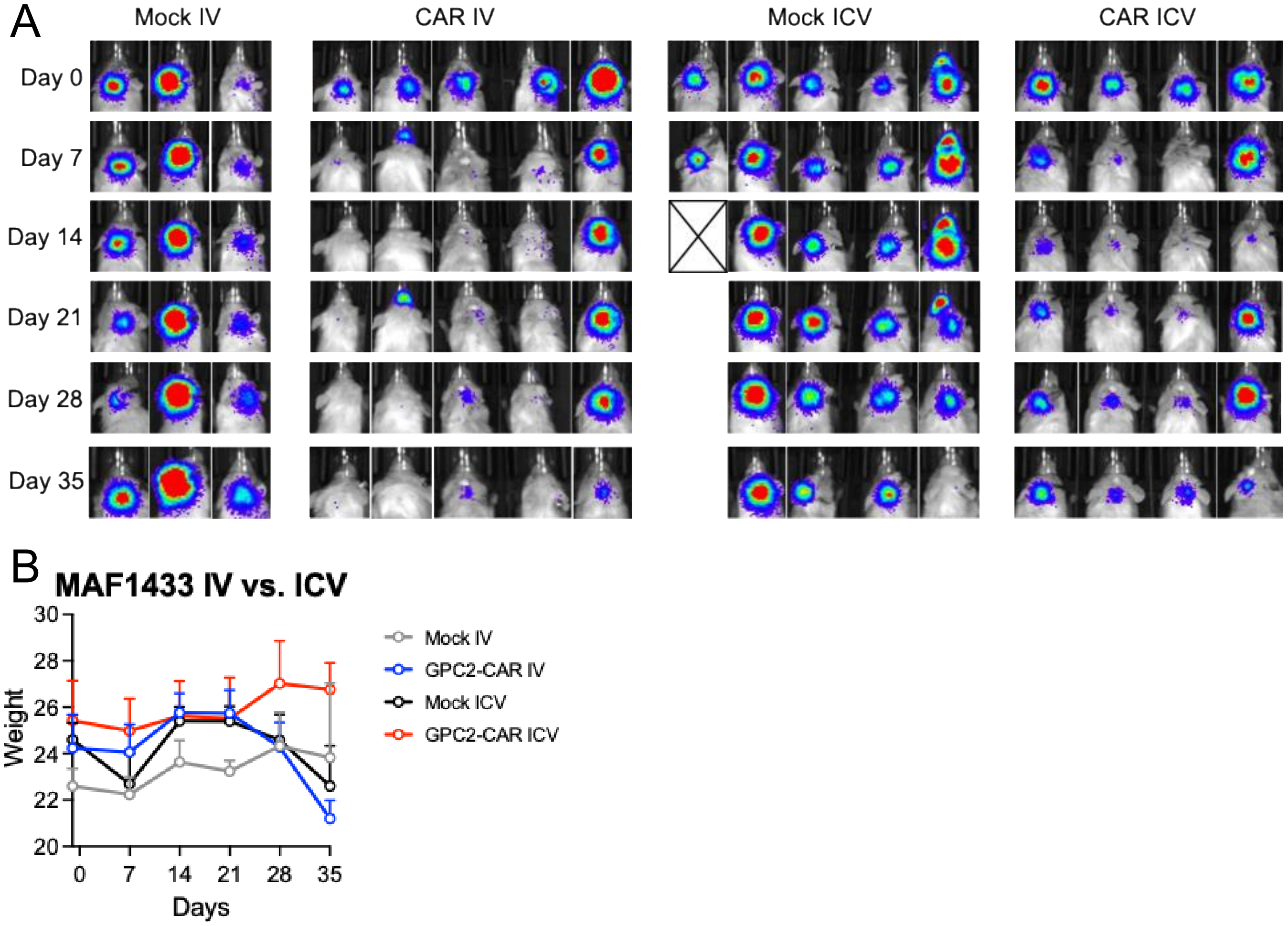

Figure S8: (A) Corresponding IVIS bioluminescence images of orthotopic MAF1433-bearing mice treated with mock or GPC2-CAR T-cells via intravenous (IV) or intraventricular (ICV) injection. **(B)** Longitudinal bodyweight measurement of study mice.

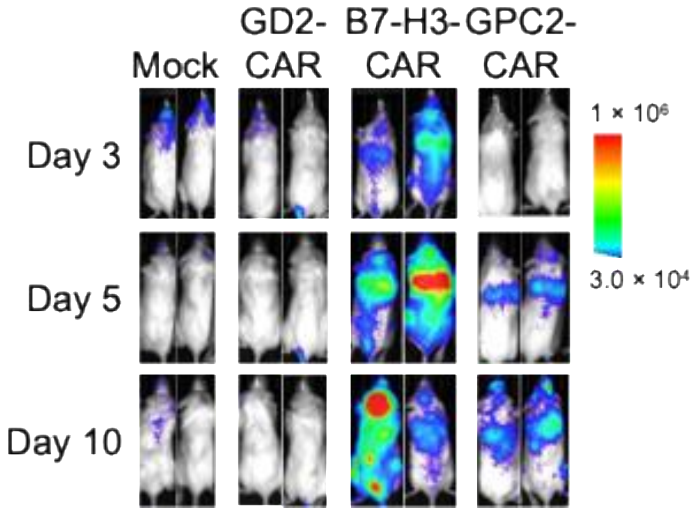

Figure S9: CAR T-cell tracking in the orthotopic D283 wildtype model. The IVIS BLI signal indicates T-cells. Over time, T-cells home to the tumor and lymphoid organs to expand.

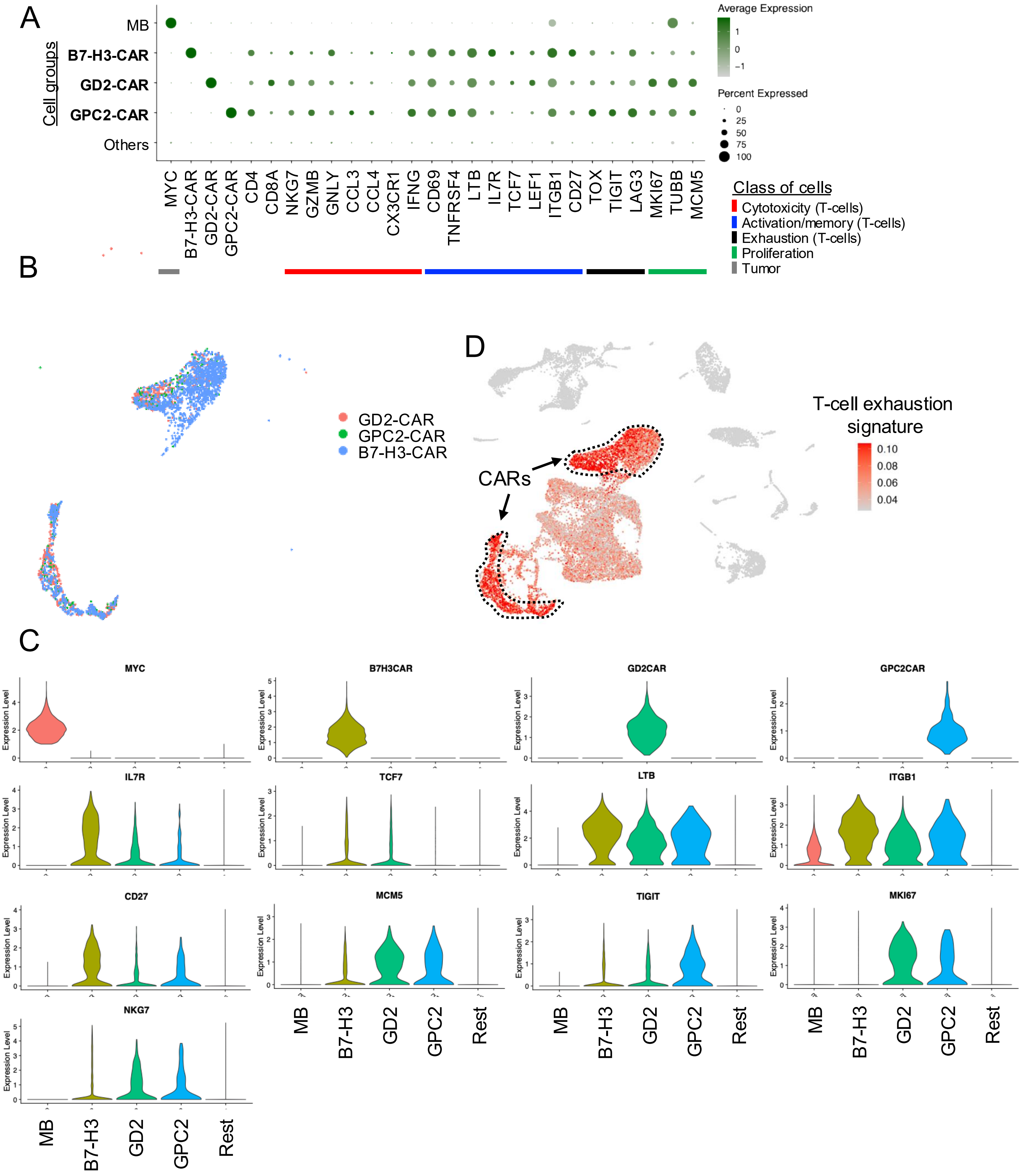

Figure S10: (A) The dot plot exhibits the relative expression of key genes organized by cellular processes. The dot size indicates the percentage of cells expressing each gene, and the dot color represents the average expression levels. **(B)** Integrated and reclustered CAR populations. **(C)** Violin plot of several key genes. **(D)** Expression levels of T-cell exhaustion gene signature in the single-cell data set.

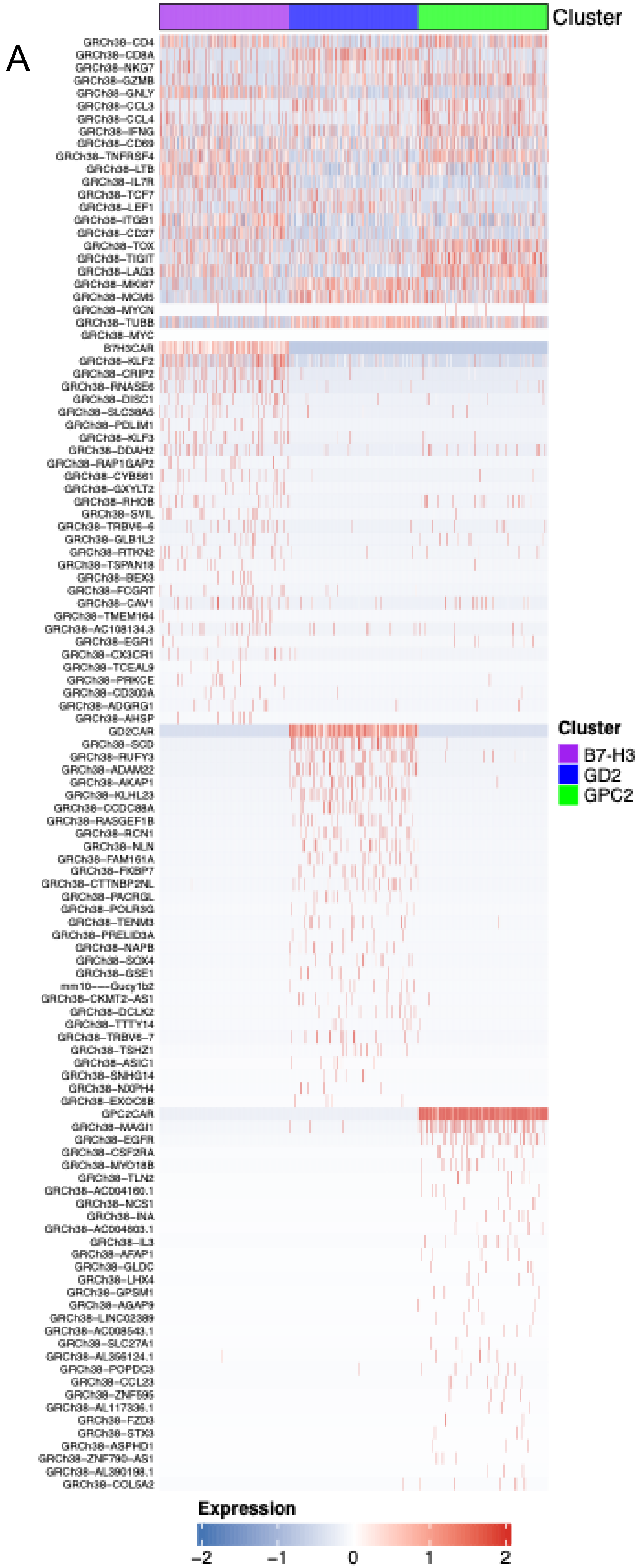

Figure S11: Heat map of top 30 genes for each CAR cluster, including a selection of genes relevant for T-cell function.

Table S1: Methylation profiles of D283 and MAF1433 categorize both lines as medulloblastoma non-WNT/non-SHH activated group 3 tumors.

	Heidelberg Epigenomics CNS Tumor Classifier	NIH Bethesda CNS Tumor Classifier
D283		
Medulloblastoma	0.906067	0.93
Non-WNT/nonSHH activated	0.825379	
Group 3	0.777417	
Subclass 2	0.662449	
MAF1433		
Medulloblastoma	0.830693	0.95
Non-WNT/nonSHH activated	0.746062	
Group 3	0.696090	
Subclass 2	0.564192	

Table S2: Pathological review of mouse cerebella post GPC2-CAR T-cell treatment.

Animal #	Treatment group	H&E, cerebellum
763	Mock IV	Tumor (2x1mm)
766	Mock IV	Tumor (2x1mm)
769	Mock IV	Tumor (1x1mm)
751	CAR IV	No tumor
752	CAR IV	No tumor
764	CAR IV	No tumor
765	CAR IV	No tumor
773	CAR IV	No tumor
754	Mock ICV	No tumor
758	Mock ICV	Tumor (2x2mm)
771	Mock ICV	Tumor (2x2mm)
772	Mock ICV	Tumor (2x2mm)
774	Mock ICV	No tumor
755	CAR ICV	Tumor (2x1mm)
756	CAR ICV	Tumor (1x1mm)
757	CAR ICV	Tumor (2x1mm)
761	CAR ICV	Tumor (1x1mm)
762	CAR ICV	Tumor (2x1mm)

IV: intravenous; ICV: intracerebroventricular

Table S3: Cluster annotation of single-cell RNA-seq data set, including cluster-defining genes.

Cluster	Species	Annotation	Differentially expressed genes
0	human	MB	*SOX4*
1	human	MB	*TTR*
2	human	B7-H3-CAR	*B7-H3-CAR, GNLY, CCL5, CD4, CD8*
3	human	MB	*SOX4, MYC, ODC1*
4	murine	M2 TAMs	*Mrc1, Apoe, Ccl8, CD68, Fcgr2b, Ccl2, Ccl7*
5	human	MB	*MYC*
6	murine	Astrocytes	*Dbi, Nnat, Rsph1, Mt3, Tmem212*
7_0	human	GD2-CAR, proliferating	*GD2-CAR, TOP2A, CCL4, CD4, CD8*
7_1	human	MB, all CARs, proliferating	*MYC, B7-H3-CAR, GD2-CAR, GPC2-CAR, CD3D*
7_2	human	GD2-CAR, B7-H3-CAR, proliferating	*B7-H3-CAR, GD2-CARCD3D*
7_3	human	B7-H3-CAR, proliferating	*B7-H3-CAR, TOP2A*
7_4	human	MB, proliferating	*MYC, TOP2A*
7_5	human	GD2-CAR, proliferating	*GD2-CAR, TOP2A*
7_6	human	GD2-CAR, proliferating	*GD2-CAR, TOP2A*
8	murine	Endothelial cells	*Bsg, Ly6c1, Itm2a, Cldn5, Flt1*
9	murine	CNS-associated macrophages	*Lyz2, Cd74, H2-Aa, H2-Ab1, H2-Eb1*
10	murine	Oligodendrocytes	*Plp1, Cryab, Aplp1, Tubb4a, Car2*
11	murine	Microglia	*Hexb, P2ry12, Cst3, Cx3cr1, Csmd3*
12	human	All CARs	*B7-H3-CAR, GD2-CAR, GPC2-CAR, CD3D, XCL1*
13	human	MB	*MLLT11*
14_0	human	B7-H3-CAR	*B7-H3-CAR, CD3D*
14_1	human	MB	*MYC*
14_2	human	MB, proliferating	*MYC, TOP2A*
14_3	human	MB	*MYC*
14_4	human	MB	*MYC*
15_0	human	GD2-CAR, proliferating	*GD2-CAR, HMGB2, TOP2A, MKI67*
15_1	human	GD2-CAR, proliferating	*GD2-CAR, HMGB2, TOP2A, MKI67*
15_2	human	B7-H3-CAR, proliferating	*B7-H3-CAR, TOP2A*
15_3	human	MB	*MYC*
15_4	human	MB, proliferating	*MYC, TOP2A*
15_5	human	MB, proliferating	*MYC, TOP2A*
16	murine	Astrocytes	*Lsamp, Atp1a2, Grid2, Atp1b2, Hsd11b1*
17	murine	PMN	*S100a9, S100a8, Retnlg, Ngp, Il1b, Lcn2, Cxcl2*
18	human	MB	*TOP2A, HMGB2, MKI67*
19	murine	Monocytes	*Plac8, S100a4, Gpx1, Lgals3, Cebpb*
20	murine	Astrocytes	*Scl1a3, Gpr37l1, Aldoc, Timp4, Plpp3*
21	murine	Astrocytes	*Dbi, Mt3, Nnat*
22	murine	Pericytes	*Vtn, Rgs5, Myl9, Acta2, Cald1*
23	murine	Antigen-presenting cells	*H2-Aa, Cd74, H2-Ab1, H2-Eb1, Ifi30, Lsp1*
24	murine	Fibroblasts	*Mgp, Igfbp6, Rbp1*
25	murine	Neurons	*Atp5g1, Ppp1r1b, Fabp3, Mdh1*
26	murine	Pericytes and endothelial cells	*Vtn, Rgs5, Igfbp7, Bsg, Ly6c1, Itm2a, Flt1, Cldn5*
27	murine	Oligodendrocytes	*Ptgds, Apod, Ptn*
28	murine	Oligodendrocytes	*Nkain2, Pcdh9, Lrp1b, Plcl1, Tmeff2*
29	murine	CNS-associated macrophages	*Bst2, Plac8, Irf8, Ly6a, Siglech*
