# Supplementary material for: GPC2-CAR T cells have potent preclinical activity against orthotopic medulloblastoma xenografts
Source: Mol Ther Oncol. 2025 Sep 30;33(4):201067. doi: 10.1016/j.omton.2025.201067 (PMC12554907; doi:10.1016/j.omton.2025.201067)
Supplement: Document S2. Article plus Supplemental information [file mmc2.pdf]

Molecular Therapy
Oncology
Original Article

GPC2-CAR T cells have potent preclinical activity against orthotopic medulloblastoma xenografts

Reona Okada,[1] Mia Fanuzzi,[1] Constanza Rodriguez,[1] Jangsuk Oh,[1] Adhithi Sreenivasan,[1] Hannah G. Stack,[1] Mariela Puebla,[1] Ira Phadke,[1] Allison P. Cole,[1] Shanshan Bradford,[2] Michael C. Kelly,[3] Haiyan Lei,[1] Mitchell Ho,[4] Jennifer A. Cotter,[5,6] Carol J. Thiele,[1] Xiyuan Zhang,[1] Anandani Nellan,[1] and Rosa Nguyen[1]

[1]Pediatric Oncology Branch, Center for Cancer Research, National Cancer Institute, National Institutes of Health, Bethesda, MD, USA; [2]Computational Biology, St. Jude Children's Research Hospital, Memphis, TN, USA; [3]Single Cell Analysis Facility, Center for Cancer Research, National Cancer Institute, Bethesda, MD, USA; [4]Laboratory of Molecular Biology, Center for Cancer Research, National Cancer Institute, National Institutes of Health, Bethesda, MD, USA; [5]Department of Pathology & Laboratory Medicine, Children's Hospital Los Angeles, Los Angeles, CA, USA; [6]Keck School of Medicine, University of Southern California, Los Angeles, CA, USA

Medulloblastoma (MB) is the most common malignant brain tumor in children. Patients with group 3 and 4 MB have poor clinical outcomes, underscoring the urgent need for new therapies. Glypican-2 (GPC2) is a recently discovered oncofetal antigen. Given its expression in brain tumors, we evaluated the preclinical activity of our GPC2-chimeric antigen receptor (CAR) against MB and compared it to two existing CARs targeting GD2 and B7-H3. Gene expression analysis of publicly available datasets was performed and validated with immunohistochemistry staining of patient samples. The MB cell line D283 and a newly generated cell line patient-derived xenograft, MAF1433, were used. Cytokine bead assays and single-cell RNA sequencing (scRNA-seq) were used for mechanistic studies. MB patient samples express up to moderate levels of GPC2. GPC2-CARs lead to significant *in vivo* tumor regression in orthotopic tumor models via intravenous or intraventricular administration route and had equivalent activity to the B7-H3-CAR against D283 and enhanced activity than GD2-CAR in both models *in vivo*. T cell kinetic studies revealed that GPC2-CAR T cells home to the area of the primary tumor, expand, and upregulate genes critical for cytotoxicity and T cell homing. These results provide a preclinical rationale for including children with GPC2^{+} MB in our upcoming clinical GPC2-CAR T cell trial.

INTRODUCTION

Medulloblastoma (MB) is the most common malignant brain tumor in children, accounting for 20% of pediatric central nervous system cancers.[1] Molecular-driven risk stratification has reshaped the therapeutic approach for MB, allowing for de-escalation of treatment in those with low-risk disease and intensification in patients with high-risk features.[2–4] Despite a 5-year overall survival of 75% with risk-adapted multimodal therapy across all MB subtypes, most affected individuals suffer from significant therapy-related late effects.[5] Furthermore, children with recurrent MB or molecular-based high-risk disease, such as those in groups 3 and 4, face a particularly dismal prognosis since treatment options are limited for this subgroup.[6,7] This underscores the urgent need for less toxic and more efficacious therapies for these children.

Chimeric antigen receptor (CAR) T cell therapy is an innovative immunotherapy that has changed the standard of care for patients with relapsed and refractory leukemia and lymphoma and shows promise in solid tumors.[8,9] Previous trials have demonstrated the feasibility of systemic and loco-regional administration of CAR T cells in children with brain tumors other than MB.[10–13] Building on these experiences, several ongoing MB-inclusive trials are targeting IL13Rα2 (NCT04510051 and NCT04661384), B7-H3 (NCT05835687 and NCT04185038), EGFR806 (NCT03638167), GD2 (NCT05298995), and HER2 (NCT03500991).

Glypican-2 (GPC2), a member of the family of heparan sulfate proteoglycans, is a *MYCN*-regulated and recently discovered oncofetal antigen expressed in various pediatric solid tumors and with limited expression in healthy tissues.[14,15] We and others have developed several distinct GPC2-CARs for patients with GPC2^{+} neuroblastoma.[16–19] Our lead CAR comprises a single-chain variable fragment (scFv), CT3, obtained through hybridoma technology, a CD28 hinge and transmembrane domain, and a 4-1BB costimulatory domain and exhibits potent preclinical activity against neuroblastoma.[17,18] Previous studies have shown that GPC2 is also expressed in

Received 20 November 2024; accepted 27 September 2025;
https://doi.org/10.1016/j.omton.2025.201067.

Correspondence: Anandani Nellan, Pediatric Oncology Branch, Center for Cancer Research, National Cancer Institute, National Institutes of Health, Bethesda, MD, USA.
E-mail: anandani.nellan@astrazeneca.com
Correspondence: Rosa Nguyen, Pediatric Oncology Branch, Center for Cancer Research, National Cancer Institute, National Institutes of Health, Bethesda, MD, USA.
E-mail: hongharosa.nguyen@nih.gov

Published by Elsevier Inc. on behalf of The American Society of Gene and Cell Therapy.
This is an open access article under the CC BY license (http://creativecommons.org/licenses/by/4.0/).

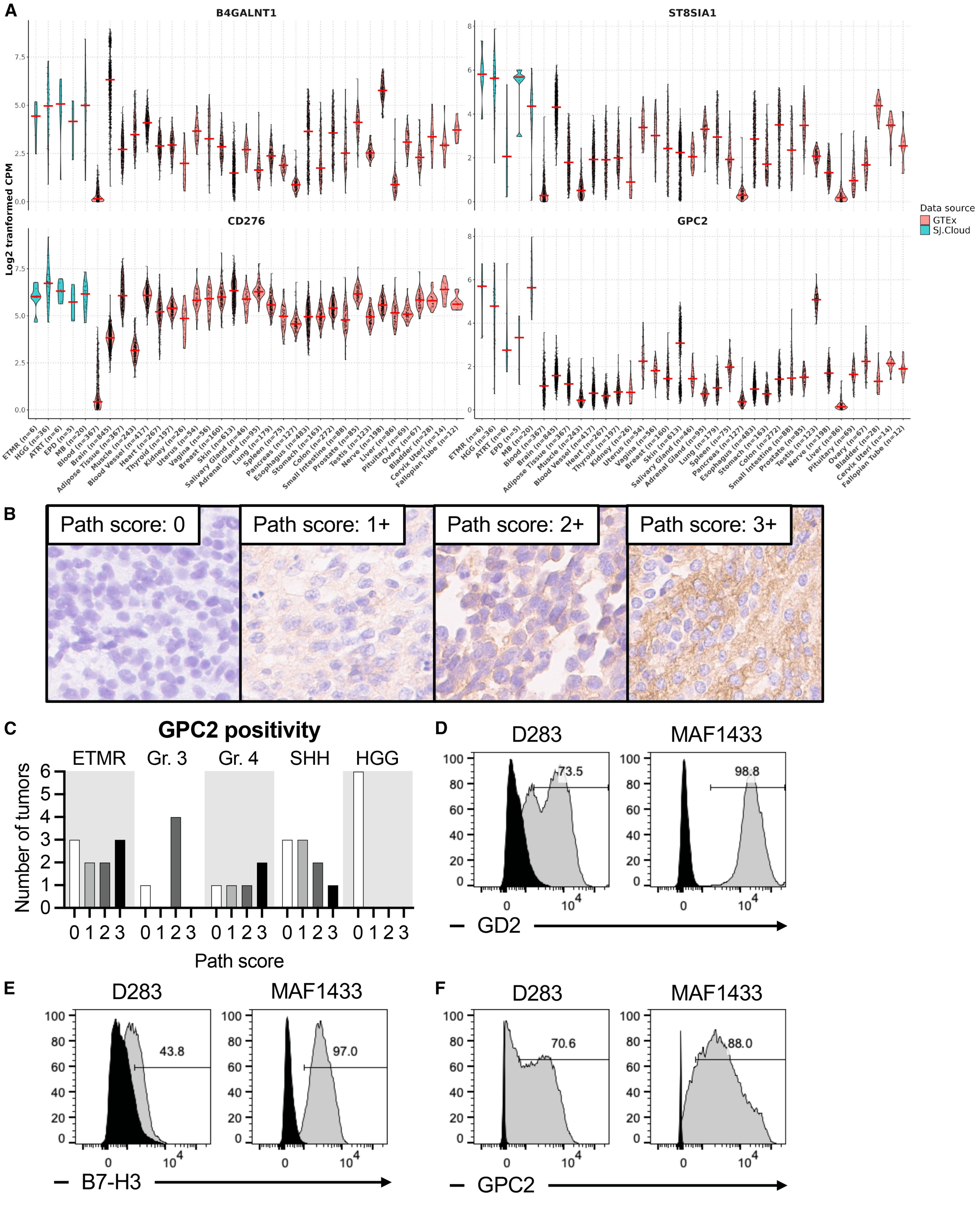

(legend on next page)

medulloblastoma.[15,20] Thus, we assessed if our GPC2-CAR possesses preclinical activity against MB by comparing it to two other clinically relevant CARs, a GD2-[21] and B7-H3-targeted CAR.[22] Using the group 3 MB cell line D283[23–25] and a newly established cell line patient-derived xenograft (PDX) from a patient with group 3 MB, MAF1433, we demonstrate that GPC2-CAR T cells effectively regressed MB *in vivo*. These results provide a rationale for including a study arm for children with GPC2$^+$ MB in our upcoming clinical GPC2-CAR T cell trial.

RESULTS

GPC2 is a CAR target in medulloblastoma

We analyzed the expression of common CAR antigens and related genes in brain tumor patient samples, PDXs, and patient-derived cell lines from the Pediatric Cancer Genome Project.[26] We found that genes responsible for GD2 synthesis (*B4GALNT1* and *ST8SIA1*) and *CD276*, encoding B7-H3, were expressed at similar or lower levels in MB than in other brain tumors. However, *GPC2* was notably upregulated in MB compared to other brain tumors and healthy tissues (Figure 1A; Figure S1). To validate these findings, we examined tumor tissues from patients with embryonal tumor with multilayered rosettes (ETMR) (n = 10), group 3 MB (n = 5), group 4 MB (n = 5), Sonic hedgehog (SHH) MB (n = 9), and high-grade glioma (n = 6), and evaluated the intensity and pattern of GPC2 immunoreactivity compared to neuroblastoma used as positive control (Figure 1B; Figure S2). GPC2 was expressed at up to moderate levels across MB subtypes (Figure 1C). In ETMR, pathology scores ranged from 0 to 3+, while high-grade gliomas showed no detectable GPC2 expression. These results are different than previous reports that used different detection antibodies and had higher intensity and positivity, particularly in high-grade gliomas.[15,20] We further measured GD2, B7-H3, and GPC2 levels by flow cytometry in two preclinical models of group 3 MB—D283 and MAF1433 (Table S1). When retrieved from cell culture, MAF1433 exhibited high proportions of cells expressing GD2, B7-H3, and GPC2 (88.0%–98.8%), while D283 cells had overall lower proportions, with B7-H3 being the lowest antigen expressed in only 43.8% of the cells (Figures 1D–1F). *In vivo*, GPC2 expression in both models was homogenous and strong (Figure S3A). These findings demonstrate that GPC2 is up to moderately expressed in patient samples and strongly expressed in preclinical models of MB, making it a suitable CAR target for immunotherapy.

GPC2-CAR T cells eliminate medulloblastoma *in vitro*

We designed CARs to target GD2, B7-H3, and GPC2 using scFvs currently in clinical development (Figure 2A).[21,27] The CAR constructs have comparable but donor-dependent CAR transduction efficiencies (Figure 2B). In 72-h co-culture cytotoxicity assays using a luciferase reporter-measuring assay to quantify cell viability, all three CARs effectively eliminated D283 (Figure 2C; Figure S4A) and MAF1433 at various E:T ratios (Figure 2D; Figure S4B). GPC2-directed CAR T cells demonstrated early and sustained activation in tumor co-culture, with increased intracellular interferon (IFN)-γ detected at 12 h and elevated granzyme B (GZMB) levels by 36 h (Figures S4C and S4D). Phenotypic analysis revealed notable differences among CAR constructs (Figure S5). Both B7-H3- and GPC2-CAR T cells exhibited minimal expression of exhaustion markers, including LAG-3, TIM-3, CD39, and PD-1, after 72 h in co-culture. These cells also maintained the highest proportions of naive and central memory phenotypes, suggesting enhanced persistence and functionality. However, when CAR T cells were repeatedly challenged with tumor cells, GPC2-CARs showed reduced cytotoxicity compared to GD2- and B7-H3-CARs, particularly against D283 cells (Figures 2E and 2F; Figures S6A and S6B). The diminished cytotoxic activity was associated with decreased GPC2 expression in the surviving tumor cell population (Figure S6C). Additionally, GPC2-CAR T cells did not exert cytotoxic effects on GPC2-negative targets, indicating that tumor cell killing is antigen-dependent (Figure S6D). Collectively, these findings support the potent but antigen-restricted cytolytic function of GPC2-CAR T cells *in vitro*.

GPC2-CAR T cells have potent cytotoxicity against medulloblastoma *in vivo*

To evaluate the activity of GPC2-CAR T cells *in vivo*, we stereotactically implanted D283 or MAF1433 into the cerebellum of NOD SCID gamma (NSG) mice (4–6 weeks old) (Figure 3A), which establishes orthotopic tumor (Figure 3B; Figures S3A and S3B). Both lines express homogeneous and strong positive levels of GPC2 *in vivo* (Figure S3A). Once the mice developed a distinct *In Vivo* Imaging System (IVIS) bioluminescence imaging (BLI) signal, they were randomized to receive untransduced mock cells or 5 × 10^6 GD2-, B7-H3-, or GPC2-CAR$^+$ T cells. In both models, treatment with GPC2- and B7-H3-CARs led to the most pronounced tumor regression by IVIS BLI (Figures 3C and 3D; Figures S7A–S7D). In the D283 model, B7-H3-CARs induced a rapid tumor decrease in tumor size by day 14 post-CAR injection, while GPC2-CARs triggered a more gradual reduction over 3–4 weeks. The converse occurred in the MAF1433 model. The GPC2-CAR induced a more rapid tumor response than the B7-H3-CAR as early as 7 days after CAR T cell injection. Ultimately, B7-H3-CAR T cells demonstrated greater efficacy, with 4 of 6 mice (67%) reaching background BLI signals, compared to 2 of 7 mice (29%) in the GPC2-CAR T cell group. In

Figure 1. GPC2 expression in medulloblastoma

(A) Expression of *B4GLANT1* and *ST8SIA1* (enzymes responsible for GD2 synthesis), *CD276*, and *GPC2* in various primary tumors, derived xenografts, and cell lines from patients with embryonal tumor with multilayered rosettes (ETMR), high-grade glioma (HGG), atypical teratoid rhabdoid tumor (ATRT), ependymoma (EPD), and medulloblastoma (MB). One-way ANOVA test with ad-hoc multiple comparisons test: ns (not significant), $p > 0.05$; *$p < 0.05$; and **$p < 0.01$. (B) Examples of pathology scores 0–3+ in brain tumor samples stained with CT3 to detect GPC2 expression. (C) Summary of pathology scoring across different tumor histologies. ETMR, group 3, group 4, and Sonic hedgehog (SHH) MB, and HGG were tested. (D–F) Flow cytometry analysis for the detection of (D) GD2, (E) B7-H3, and (F) GPC2.

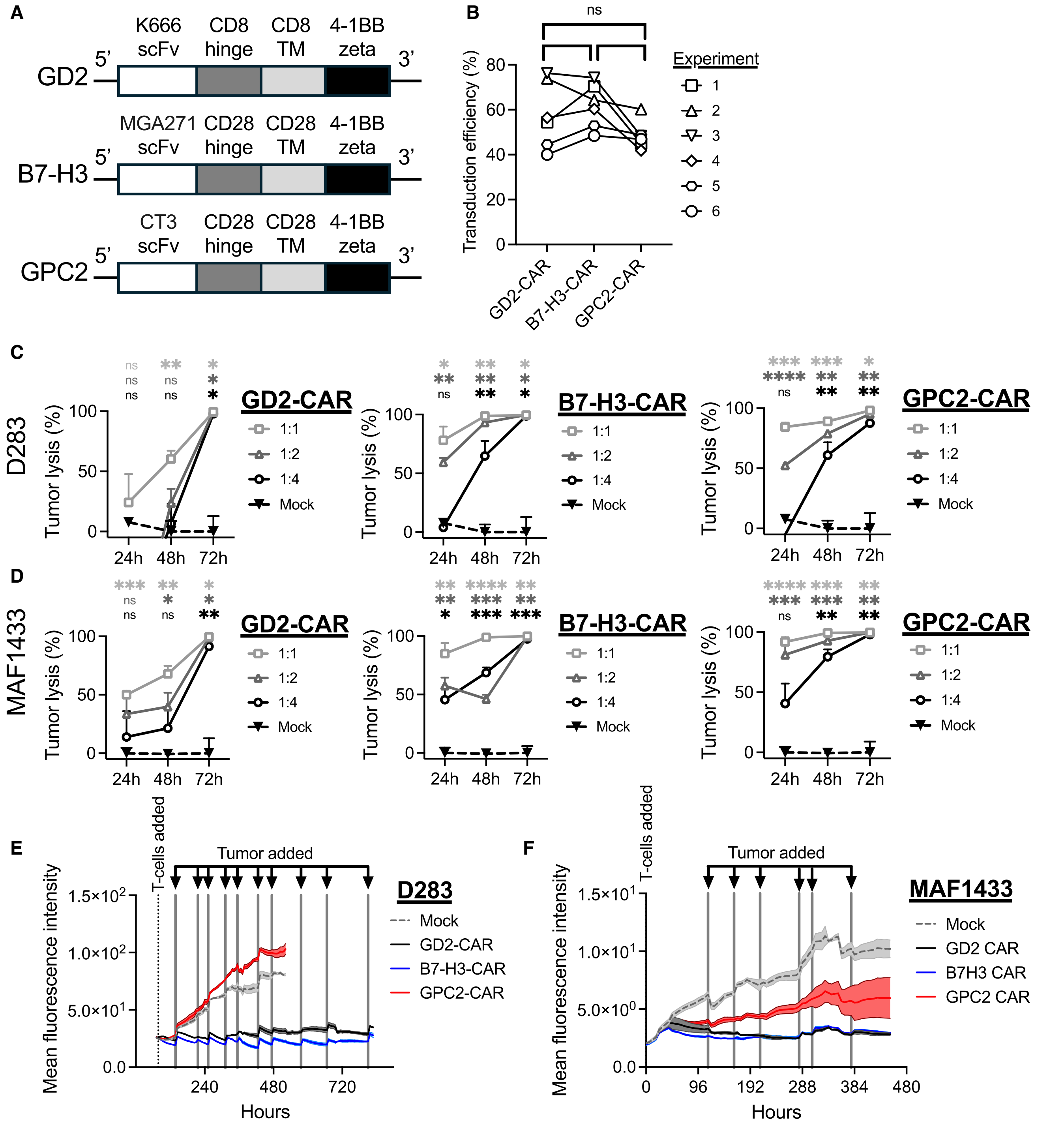

Figure 2. *In vitro* testing of GPC2-, B7-H3-, and GD2-CARs against medulloblastoma

(A) Schematic of the GD2-, B7-H3-, and GPC2-CAR constructs used in this study. (B) Transduction efficiency of T cells transduced with the construct in (A). (C and D) Seventy-two-hour *in vitro* cytotoxicity assay of GD2-, B7-H3-, and GPC2-CAR against (C) D283 and (D) MAF1433. The OneGlo luciferase-based assay was used to quantify viable cells. One of two replicates is shown with technical triplicates per condition. One-way ANOVA test with ad-hoc multiple comparisons test. ns, not significant; $*p < 0.05$; $**p < 0.01$; and $***p > 0.001$. (E and F) Tumor rechallenge assay against (E) D283 and (F) MAF1433. Tumor rechallenges are indicated by the lines and arrows. The total green object intensity is measured to quantify GFP^+ tumor viability. One-way ANOVA test with ad-hoc multiple comparisons test. $****p < 0.0001$. Means and standard deviations are plotted in subpanels (C)–(F).

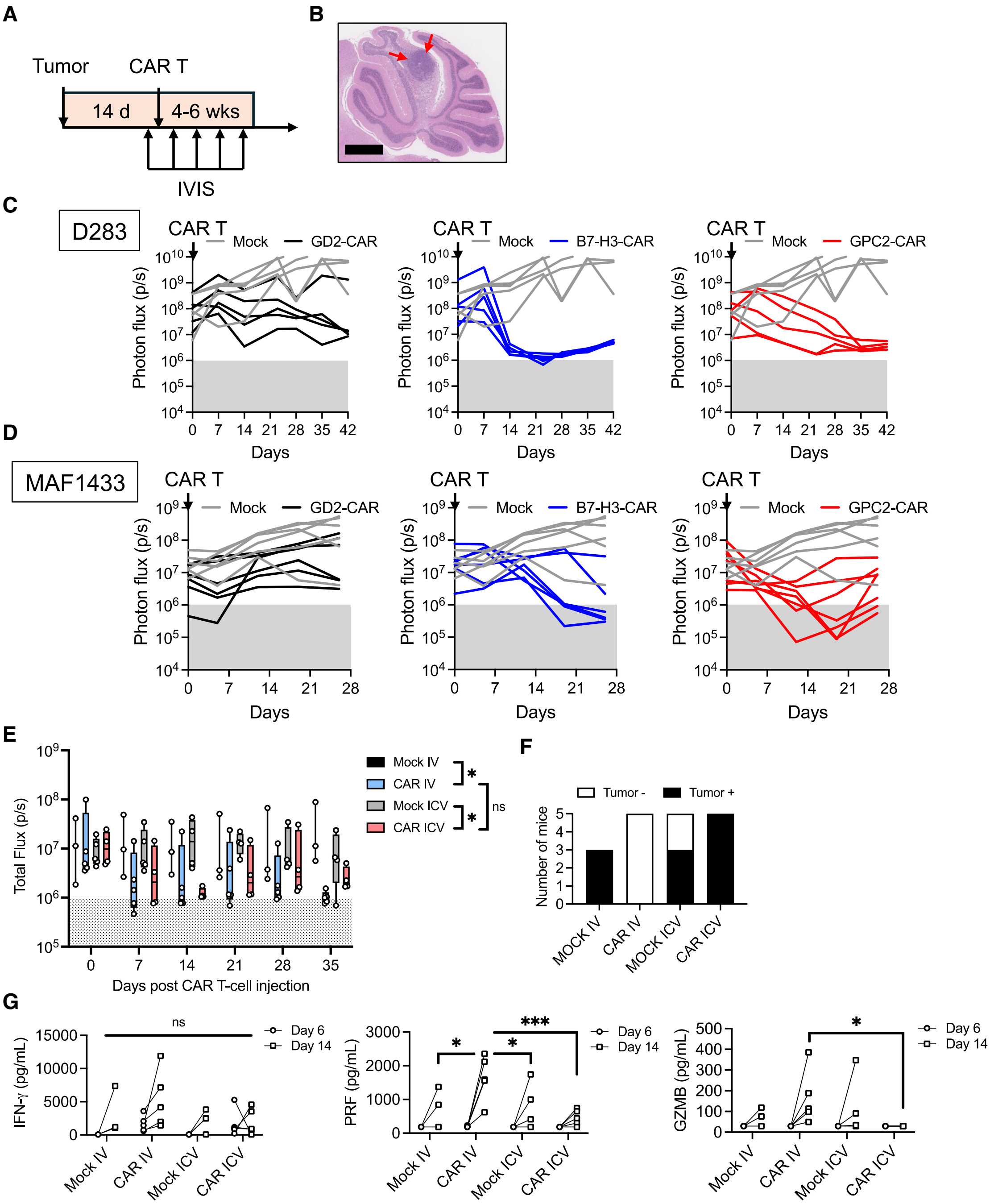

Figure 3. GPC2-CAR T cells induce potent tumor regression against medulloblastoma *in vivo*

(A) Experimental schema of *in vivo* studies. After stereotactic tumor injections, mice are given 14 days for tumor engraftment before undergoing IVIS BLI for randomization. CAR T cell injection occurs on day 0. Weekly IVIS BLI is performed for tumor tracking. (B) H&E staining demonstrates a representative orthotopic tumor location after stereotactic injection into the cerebellum. Scale bars, 1 mm. (C and D) IVIS BLI signals of tumor burden after CAR T cell therapy for (C) D283 and (D) MAF1433. (E) Comparison of intravenous (i.v.) and intraventricular (ICV) administration of GPC2-CAR T cells to treat MAF1433-bearing mice. Two-way ANOVA test with ad-hoc multiple comparisons test. *$p < 0.05$; ns $p > 0.05$. (F) Histological tumor status at the end of the study of mice from (E). (G) On day 6 and day 14, animals underwent serum analysis of cytokines, including IFN-γ, perforin (PRF), and granzyme B (GZMB). One-way ANOVA test with ad-hoc multiple comparisons test. *$p < 0.05$; ***$p < 0.001$; and ns $p > 0.05$. Means and standard deviations are plotted in subpanel (E).

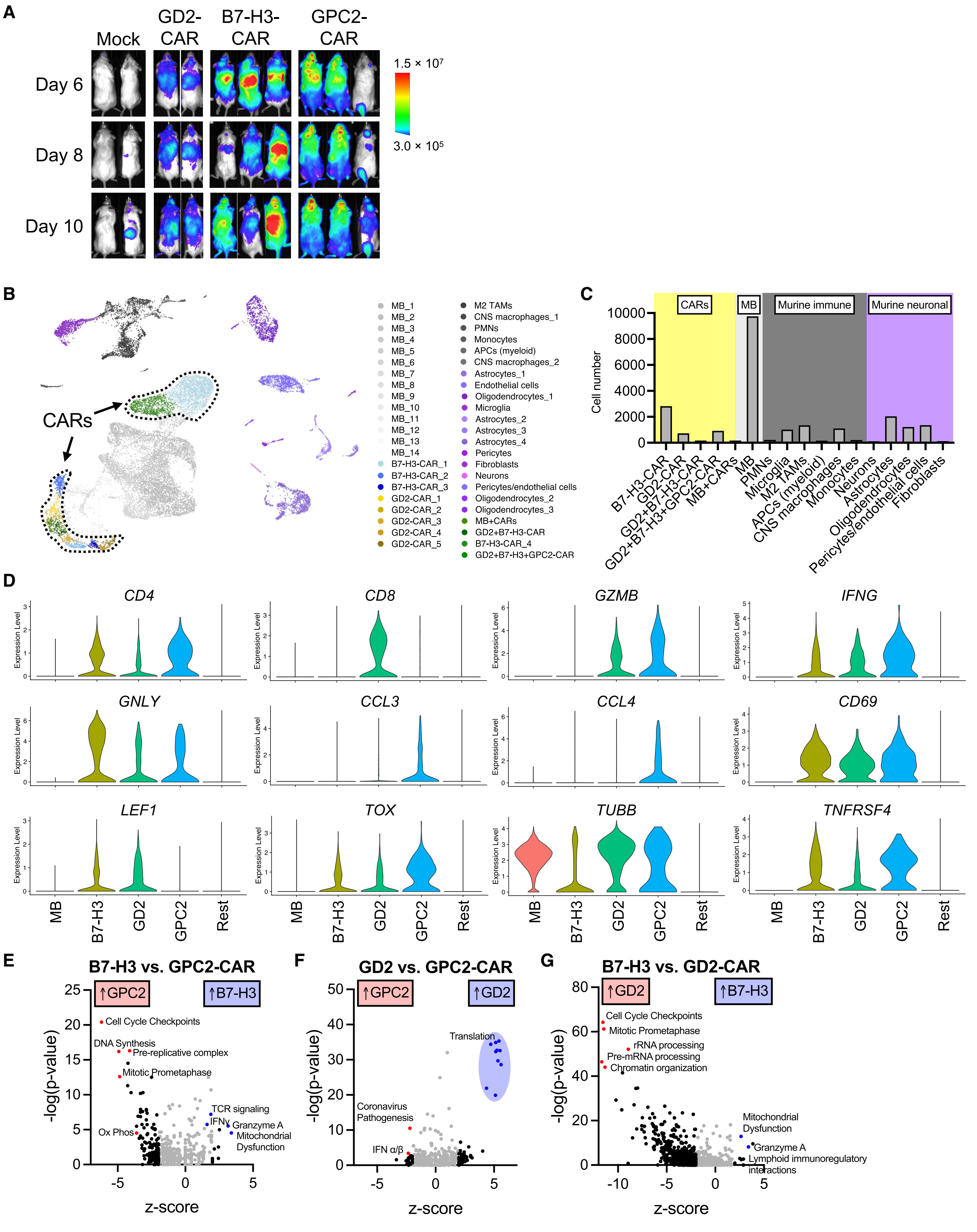

(legend on next page)

the latter group, histological review detected tumor cells in the cerebellum of only two mice with marked downregulation of GPC2 expression (Figure S7E).

Recent clinical studies demonstrate that locoregional administration of CAR T cells can be well tolerated without the requirement for lymphodepleting chemotherapy in patients with brain tumors.[28,29] To identify the most effective translational strategy for GPC2-targeted CAR T cell therapy, we compared intracerebroventricular (ICV) and intravenous (i.v.) delivery routes in the orthotopic MAF1433 model. Following engraftment with the MAF1433 cell line, mice were administered 2×10^6 CAR-positive T cells via either i.v. or ICV injection. BLI using IVIS revealed significantly reduced signal intensity in both treatment groups relative to controls receiving mock T cells, indicating tumor regression (Figure 3E; Figure S8A). However, no statistically significant difference in BLI signal was observed between i.v. and ICV CAR T cell-treated groups. To assess residual tumor burden, cerebellar tissue was examined histologically at the study endpoint. Consistent with BLI findings, no residual tumor was detected in mice treated via i.v. delivery, whereas all animals in the ICV group exhibited microscopic disease (Figure 3F; Table S2). Importantly, animals receiving GPC2-CAR T cells via ICV administration maintained stable body weight throughout the study, suggesting improved tolerability with this route (Figure S8B). Supporting this observation, serum levels of perforin and GZMB were elevated in animals treated intravenously compared to those treated ICV (Figure 3G). These results indicate that both delivery routes effectively targeted orthotopic MAF1433 tumors; however, i.v. administration resulted in a higher rate of complete tumor regression. Despite this enhanced efficacy, i.v.-treated animals showed increased systemic proinflammatory cytokines and developed weight loss toward the end of the study, indicative of greater systemic toxicity. In contrast, the ICV group exhibited better clinical stability, highlighting a potential trade-off between efficacy and tolerability. Together, these findings highlight the potency of GPC2-CAR T cells against $GPC2^+$ MB *in vivo*. To further characterize the profile of MB-infiltrating CAR T cells, we employed single-cell RNA sequencing (scRNA-seq).

Cytotoxic CD4 GPC2-CAR T cells dominate in the tumor microenvironment (TME) in medulloblastoma during peak infiltration

To gain a deeper understanding of the molecular characteristics of GPC2-CAR effector cells at peak tumor infiltration when tumor regression has not fully occurred yet, we performed tracking experiments to examine the kinetics and spatial distribution of luciferase-expressing CAR T cells post-injection in MB-bearing mice. After tail vein injection, CAR T cells initially accumulated in the lungs, then migrated to the tumor site and lymphoid tissues for expansion (Figure 4A; Figure S9). By day 10 post-transfer, the IVIS signal increased at the tumor site by at least one log-fold in some of the conditions, prompting us to isolate effector and tumor cells for further profiling of the early immunological changes by scRNA-seq analysis.

We captured a total of 23,443 cells, which formed 45 distinct clusters (Figure 4B). Based on differentially expressed genes, we manually annotated each cluster (Table S3; Figures S10A). The largest cell cluster was composed of tumor cells, followed by CAR T cells, among which B7-H3-CAR T cells being the most abundant (1,693 cells), followed by GD2-CARs (891 cells) and GPC2-CARs (231 cells; Figure 4C). To compare the transcriptome across the three CARs, we isolated, integrated, and reclustered CAR-expressing cells (Figures S10B). B7-H3 and GPC2-CAR T cells were mainly CD4-positive, while GD2-CAR T cells were CD8-positive (Figure 4D). GPC2-CAR T cells exhibited the highest expression of *GZMB*, *IFNG*, and chemokines involved in $CD8^+$ effector recruitment, including *CCL3* and *CCL4* (Figure 4D). These cells were highly activated (e.g., *CD69* and *TNFRSF4*) and proliferative (e.g., *TUBB* and *MKI67*), while expressing low levels of T cell memory genes (e.g., *LEF1*, *IL7R*, and *TCF7*) but high levels of immune checkpoint and exhaustion-associated genes, such as *TOX* (Figure 4D; Figure S10C). Analysis of exhaustion gene signatures[30] revealed a clear enrichment within the CAR T cell clusters, consistent with antigen stimulation (Figure S10D). Further evaluation of CAR clusters using ingenuity pathway analysis revealed that GPC2-CAR T cells had an enrichment in pathways related to cell replication compared to the other two CAR T cells. GD2-CARs were enriched in pathways of cell replication and mRNA processing compared to B7-H3-CARs and translation compared to GPC2-CARs (Figures 4E–4G; Figure S11). Interestingly, B7-H3-CARs revealed upregulation of granzyme A, IFN-γ, and mitochondrial dysfunction pathways, indicating that their transcriptional program was committed to cytotoxicity on day 12 post-CAR injection. These observed transcriptional changes during early infiltration and expansion in all cell clusters are consistent with the *in vivo* kinetics of CAR T cell killing, which showed tumor regression to occur first in the GPC2-CAR group as early as 7 days after adoptive cell transfer, while by B7-H3-CAR and GD2-CAR groups have the most pronounced killing around 14 days (Figures 3D). These data also revealed that the anti-MB response in GPC2-CAR T cell-treated mice on day 12 was CD4-driven and marked by upregulation of key genes involved in proliferation, cytotoxicity, and CD8 effector cell recruitment.

DISCUSSION

Our study shows that MB patient samples express up to moderate levels of GPC2 and can be targeted with a GPC2-CAR, leading to significant *in vivo* tumor regression in orthotopic cell line and PDX cell line models. T cell kinetic studies revealed that GPC2-CAR T cells

Figure 4. Single-cell RNA-seq analysis of tumor-infiltrating CAR T cells at peak infiltration

(A) Tracking of T cells in MAF1433-bearing mice. T cells are luciferase-expressing and imaged on days 6, 8, and 10 post-CAR injection. (B) UMAP of the integrated tumor, tumor-infiltrating human and murine immune, and stromal cells from all samples. The independent clustering yielded 45 clusters. (C) Frequencies of cells based on manual annotations. (D) Violin plots show the expression levels of common genes across the different CARs, tumor cells, and the rest of the cells. (E–G) Ingenuity pathway analysis of differentially expressed genes comparing (E) GPC2- vs. B7-H3-CAR T cells, (F) GPC2- vs. GD2-CAR T cells, and (G) GD2- vs. B7-H3-CAR T cells.

home to the area of the primary tumor, expand, and upregulate genes critical for cytotoxicity and T cell recruitment. Altogether, these results show that GPC2-CAR T cells have cytotoxic activity against GPC2-expressing MB and offer a rationale for including a study arm for children with $GPC2^{+}$ MB in our upcoming clinical GPC2-CAR T cell trial.

To give our studies more clinical context, we performed head-to-head comparisons of the GPC2-CAR against two other clinically relevant targets—GD2 and B7-H3—for which there are ongoing trials for patients with MB (B7-H3: NCT05835687 and NCT04185038; GD2: NCT05298995). We found that GPC2-CAR T cells had equivalent to enhanced activity compared to the B7-H3- and GD2-CAR *in vivo*. Interestingly, the response kinetics of each CAR were different, with GPC2-CAR T cells inducing the most rapid tumor regression within the first 7 days in the MAF1433 model. scRNA-seq results of tumor-infiltrating T cells on day 12 in MAF1433 demonstrated that B7-H3-CAR T cells were the most enumerated cells among the effectors and transcriptionally committed to cytotoxicity as evidenced by the upregulation of key cytotoxicity pathways. GPC2-CAR T cells comprised the lowest proportion at that time, possibly because tumor clearance has already occurred. Significantly upregulated pathways in GD2-CAR T cells regulate chromatin organization, RNA processing, and cell cycle, but genes of cytotoxicity were overall expressed at lower levels compared to the other CARs, which aligns with the lower cytotoxicity *in vivo* of GD2-CARs. Although all three CARs contain a 4-1BB costimulatory domain, they were designed with different hinge and transmembrane domains to optimize their cytotoxic function.[18] These differences may contribute to the distinct transcriptional profiles during early effector activation.[16,18] It is also possible that the scFv affinity and antigen density on the tumor cells influence the homing properties and proliferative capacity of the CAR T cells. These molecular aspects warrant further investigation in the future. To identify the most effective translational strategy for GPC2-targeted CAR T cell therapy, we also compared ICV and i.v. delivery routes. Administering the same cell dose for each treatment group, both i.v. and ICV administration of GPC2-directed CAR T cells effectively regress orthotopic MAF1433 tumors. However, i.v. administration appears to confer superior efficacy in achieving complete tumor regression, despite potential concerns for toxicity. However, given the limitations of using an immunocompromised mouse model, direct translation of these findings to the clinical setting may be challenging. Therefore, validation of these results in human studies will be essential.

We validated results from a previous study, in which mRNA electroporation was used to transiently express GPC2-CARs to target $GPC2^{+}$ high-grade glioma and MB.[20] Similar to Foster et al., we found that our GPC2-CAR was active against $GPC2^{+}$ MB despite using a different scFv, method to induce CAR expression, and model systems. Interestingly, we did not detect GPC2 in high-grade gliomas using our scFv, CT3, which binds to the cancer-specific exons 3 and 10 of GPC2. Previous studies used the F-5 clone to detect GPC2.[15,20] This clone detects an epitope mapping between amino acids 450–473 in exon 9 and may explain the differences between staining patterns and detection.[17] Future quantification and correlation with CAR efficacy should be performed to better define the relationship between GPC2 levels and GPC2-CAR cytotoxicity. In the previous study, an important rationale for conducting mRNA transfection is that transient GPC2-CAR expression could mitigate potential toxicities. In our study, GPC2-CAR T cell-treated mice tolerated treatment and had elevated levels of immune-suppressive interleukin-10 in their serum after tumor clearance, though these observations in xenograft mouse models may not accurately predict on-target off-tumor toxicities in the clinic. The previously reported lack of cross-reactivity of the CT3 scFv with healthy tissues except male testis[17] may positively influence the tolerability of GPC2-CARs in humans. Ultimately, clinical testing will be essential to understand the feasibility and tolerability of GPC2-CAR (CT3-CD28HTM-BBζ) in humans.

Our *in vitro* and *in vivo* experiments demonstrate that antigen downregulation can reduce the ability of CAR T cells to eliminate tumors. This observation underscores the importance of a comprehensive approach incorporating *in vitro* and *in vivo* models when evaluating the preclinical activity of CAR T cells and understanding mechanisms of CAR T cell resistance.[31] We established a new group 3 MB cell line PDX for our studies. Compared to the D283 cell line model, our cell line PDX was more resistant to CAR killing *in vivo* despite having higher antigen levels for GPC2, GD2, and B7-H3, suggesting that this cell line PDX may possess additional unique resistance factors that are lacking in immortalized cell line models.

Lastly, we acknowledge that our study has limitations. We observed low CAR T cell counts in our single-cell samples, as we did not further enrich for T cells to preserve overall sample quality. This limitation may affect the statistical power of our analyses. Furthermore, it is unclear if locoregional administration is superior for all primary brain tumor entities or is limited to cancer types with impaired T cell invasion and a dysfunctional blood-brain barrier. The optimization of CAR T cell delivery will require future studies. We used immunocompromised NSG mice in our study, which limits our ability to fully investigate mechanisms related to CAR killing and resistance due to the lack of bystander immune cells and long-term follow-up studies due to the occurrence of xenogeneic graft-versus-host disease.[32] Studies related to these aspects may be conducted in the future when syngeneic GPC2/CAR models become available.

In summary, we showed that the CT3-CD28HTM-BBζ GPC2-CAR can regress $GPC2^{+}$ MB preclinically, providing a scientific rationale for including children with $GPC2^{+}$ MB in our upcoming clinical GPC2-CAR T cell study. Careful design of correlative studies may allow us to answer critical questions related to CAR and GPC2 biology that cannot be studied in the laboratory due to the inherent limitations of currently available preclinical models.

MATERIALS AND METHODS

Cell lines

The MB cell line D283 and a newly generated cell line PDX MAF1433 were used in this study. D283 was purchased from ATCC (HTB-185)

and maintained in full DMEM media (Lonza) with 10% heat-inactivated fetal bovine serum (FBS), 100 international units (IU)/mL penicillin, 100 μg/mL streptomycin, and 2 mM L-glutamine (Gibco). The PDX was generated by implanting patient tumor cells into (NSG mice and consecutively passaging in mice. For *in vitro* assays, MAF1433 was maintained on Matrigel-coated dishes (Corning) in FBS-free DMEM/F12 (1:1) neurobasal media with B27 supplement (Gibco) and 20 ng/mL recombinant human epidermal growth factor (PeproTech) and 40 ng/mL recombinant human fibroblast growth factor (R&D Systems). The cells were tested for mycoplasma before use (MycoAlert, Lonza), and cell identity was confirmed by short-tandem repeat DNA profiling. Luciferase-eGFP-expressing cells were created by lentiviral transduction and subsequent selection with 0.5 μg/mL of puromycin (Thermo Fisher Scientific, D283) or fluorescence-activated sorting (MAF1433).

CAR constructs and CAR T cell manufacturing

The structure and biological properties of our GPC2-CAR were previously published.[18] The codon-optimized scFv sequences of a GD2-(K666-CAR)[21] and B7-H3-targeted CAR[22] were used to engineer CAR constructs by cloning into the pELNS third-generation self-inactivating lentiviral expression vector. The CAR vectors contain a human CD8 or CD28 hinge and transmembrane domains, human 4-1BB-CD3ζ signaling endo-domain, and are expressed under an EF-1α promoter. To generate CAR T cells, cryopreserved human T cells from healthy volunteer donors (NIH Blood Bank) were transduced, activated, and expanded *ex vivo* as previously described.[31] For T cell tracking experiments, T cells were co-transduced with a luciferase-eGFP lentiviral vector (Addgene). T cells were grown in culture until day 8–10 for subsequent experiments. One donor per experiment was used to manufacture CAR T cells when multiple CARs were used for head-to-head comparisons.

***In vitro* CAR T cell cytotoxicity assay**

CAR T cell cytotoxicity was tested using a CAR T/MB co-culture system with variable effector-to-tumor (E:T) ratios. Tumor viability was assessed via live-cell imaging (IncuCyte, Sartorius) or the ONEGlo luciferase assay (Promega) to quantify luciferase-reporter gene expression as a measure of cell viability.[18] For tumor rechallenges, tumor cells were added to the plate every 24–48 h. The confluence of luciferase-eGFP-expressing tumor cells was compared among experimental conditions.

***In vivo* medulloblastoma/CAR T cell model**

All animal studies were approved by the NIH Institutional Animal Care and Use Committee (IACUC). D283 (0.5×10^6) or MAF1433 cells (0.25×10^6) were stereotactically injected in the cerebellum of NSG mice (4–6 weeks old), using established coordinates (*x*: 2 mm lateral, *y*: −3 mm caudal, and *z*: −2 mm dorsal from lambda). Typically, 100% of mice injected with D283 or MAF1433 cells engraft with tumors. For studies administering CAR T cells via the ICV route, we implanted an IVIS-compatible brain catheter (Alzet, cat. no. 0008663) one week after stereotactic tumor injection as previously described.[33] Two weeks after tumor injection, mice underwent IVIS BLI and were randomized to receive either $2–5 \times 10^6$ CAR$^+$ T cells or matched total numbers of untransduced mock T cells. For i.v. tail vein injections, cells were resuspended in 100 μL of PBS. For ICV injections, cells were administered in 5 μL via the intracranial catheter. Animals were followed weekly with IVIS BLI for 4–6 weeks or taken off the study earlier if they exhibited neurological symptoms, clinical decline, or ≥15% weight loss. At the end of the study, cerebella and spleens were collected for further analysis. Serum was collected on days 6, 14, and 35 for cytokine analysis.

***In vivo* CAR T cell tracking**

NSG mice (4–6 weeks old) were injected with D283 or MAF1433 WT cells as described previously. Based on known tumor growth kinetics, 5×10^6 CAR$^+$ T cells were injected intravenously at approximately 3 weeks post-tumor injection. IVIS BLI was used every 2–4 days to track luciferase-eGFP-expressing CAR T cells *in vivo*.

Immunohistochemistry

Paraffin-embedded tumors from patients with therapy-naive MB ($n = 18$), high-grade glioma ($n = 6$), or ETMR ($n = 10$) were dewaxed. Antigen was retrieved prior to incubation with a murine anti-human GPC2 antibody (CT3, Bioxcell) at a concentration of 1 μg/mL and subsequently secondary anti-mouse antibody. J.A.C. performed pathological scoring (scores from 0 to 3+).

Flow cytometry

About 1×10^6 cells were stained at room temperature for 12 min with antibodies against GD2 (all BioLegend; clone: 14.G2a), B7-H3 (MIH42), or GPC2 (CT3). Cells were washed twice with flow buffer (PBS + 2% FBS), and data were ultimately acquired on an LSRFortessa (BD Biosciences). For intracellular staining of T cells, we used the eBioscience Foxp3/Transcription Factor staining Buffer Set (Protocol B) for permeabilization and fixation and followed the manufacturer's instructions. Antibodies against CD8 (all BioLegend; clone: HIT8a), CD4 (SK3), IFN-γ (W19227A), and GZMB (QA16A02) as well as G4S (Cell Signaling Technology; E7O2V) were used. Flow cytometry data were further analyzed with FlowJo (version 10.10.0).

Cytokine bead analysis

Serum was collected at the end of the study to quantify common cytokines in treated mice. Following the manufacturer's protocol, we conducted cytokine bead assays (BioLegend). Data were analyzed in GraphPad Prism (version 10.3.1).

RNA-sequencing analysis

Publicly available data from the Pediatric Cancer Genome Project[26] and GTEx were obtained and re-analyzed in this study. RSEM (v.1.3.0) coupled with Bowtie2 (v.2.2.9) was used to quantify the expression of genes and transcripts with gene annotation from GENCODE (release v.32). For analysis of *GPC2* expression, raw data were realigned to include cancer-specific exons 3 and 7–10.

Raw transcript-level counts with and without gene-level counts were used for comparisons of expression levels.

Single-cell RNA sequencing

Cerebella from 3 to 4 mice per therapy group were dissociated and pooled. The scRNA-seq samples were processed with the 10X Genomics 5′ v2 chemistry kit for library generation following the manufacturer's protocol. Our goal was to capture up to 10,000 cells per lane. The cDNA library was sequenced on the Illumina NovaSeq 6000 platform, with a target depth of approximately 50,000 reads per cell.

Single-cell RNA sequencing analysis

Base calling was performed using RTA v3.4.4. before demultiplexing using CellRanger v7.2.0 (10× Genomics, Bcl2fastq 2.20.0). The resulting FASTQ files were aligned to a custom human GRCh38 and mouse mm10 reference sequence with the binding domain sequences of CARs, using CellRanger v7.2.0 (STAR v.2.7.2a). Both raw and filtered gene-barcode matrices were created with CellRanger and further analyzed in Seurat (v.5.1.0, R package).[34] Ambient RNA was removed with SoupX,[35] and doublets with scrublet.[36] Low-quality cells with <300 genes, >7,500 genes, >70% of reads mapped to mitochondrial genes, <500 unique molecular identifiers (UMI), or >50,000 UMI were removed. The data was integrated across different samples with the reciprocal principal-component analysis (RPCA) in Seurat.[37] To normalize the data, we used the "NormalizeData" function with the following parameters: normalization.method = "LogNormalize" and scale.factor = 10,000. The "FindVariableFeatures" function with "vst" method was applied to identify 3,000 highly variable genes. The "ScaleData" function with default parameters was used to scale and center gene expression matrices. To create PCA plots, we ran the "RunPCA" function. The shared nearest neighbor (SNN) graph was created with the "FindNeighbors" function. We determined the clusters by applying the Louvain algorithm via the "FindClusters" function, with a resolution set to 0.8. Manual cluster annotation was done by examining the top-ranked differentially expressed genes using the "FindAllMarkers" function. CAR T cells were identified when the cells had more than 0 reads aligned to the corresponding binding domain sequence. Differential gene expression was calculated among all clusters. Data was visualized through uniform manifold approximation and projection (UMAP).

Statistical analysis

Data distribution was examined with D'Agostino-Pearson omnibus and Shapiro-Wilk normality tests. When more than two groups were compared, we used one-way analysis of variance (normal distribution) and Tukey's post-hoc comparison tests. For skewed data, we log-transformed the data and/or used non-parametric tests (Kruskal-Wallis tests with Dunn post-hoc analysis). A p value <0.05 was considered statistically significant. Statistical analyses were performed using the GraphPad Prism version 10.

DATA AND CODE AVAILABILITY

The data are available from the corresponding author upon reasonable request. The scRNA-seq data were deposited in the GEO database (GSE281233).

ACKNOWLEDGMENTS

This work was supported by the Center for Cancer Research (CCR; ZIA BC 012066 to R. N.), the Solving Kids Cancer Foundation, the Bibi Fund for Rare Childhood Cancer Research, and Princess Nora's Warriors Foundation (R.N., A.N., and C.J.T.). The PDX was established with support from the Morgan Adams Foundation at the University of Colorado Anschutz Medical Campus. The CCR single-cell analysis facility was funded by the FNLCR contract 75N91019D00024. The computational resources of the NIH HPC Biowulf cluster (http://hpc.nih.gov) were used in this study. We thank the NCI CCR Animal Resource Program/NCI Biological Testing Branch (Dr. M. Custer and Kathy Divi) for providing mice, the NIH CC Blood bank (Thomas Lewis) for providing healthy donor blood cells, the Biological Resources Branch at the Frederick National Laboratory for giving us cytokines, Dr. A. Mendoza for his assistance with animal regulatory work, J. Buckley for his assistance with animal work, and Y. Sakhalkar for reading the manuscript. Human tumor biospecimens were provided by the USC Norris Cancer Center Translational Pathology Core (P30CA014089) and the CHLA Center for Pathology Research Services.

AUTHOR CONTRIBUTIONS

R.O.: formal analysis, data curation, visualization, and writing of the original draft; M.F., C.R., J.O., A.S., H.G.S., M.P., I.P., and A.P.C.: data curation and review/editing of the draft; S.B. and H.L.: data analysis and review/editing of the draft; M.C.K. and M.H.: methodology and review/editing of the draft; J.A.C.: pathology review and review/editing of the draft; C.J.T.: review/editing of the draft and funding acquisition; X.Z.: methodology, formal analysis, data visualization, and review/editing of the draft; A.N.: conceptualization, methodology, supervision, and review/editing of the draft; R.N.: conceptualization, methodology, formal analysis, data curation, visualization, supervision, writing of the original draft, and funding acquisition.

DECLARATION OF INTERESTS

M.H., C.J.T., and R.N. filed a patent related to GPC2-CAR (no. 20250144214).

SUPPLEMENTAL INFORMATION

Supplemental information can be found online at https://doi.org/10.1016/j.omton.2025.201067.

REFERENCES

1. Ries LAG, Y.J., Keel, G.E., Eisner, M.P., Lin, Y.D., and Horner, M.-J. (2007). SEER Survival Monograph: Cancer Survival Among Adults: U.S. SEER Program, 1988-2001, Patient and Tumor Characteristics (National Cancer Institute). SEER Program.

2. Gajjar, A., Robinson, G.W., Smith, K.S., Lin, T., Merchant, T.E., Chintagumpala, M., Mahajan, A., Su, J., Bouffet, E., Bartels, U., et al. (2021). Outcomes by Clinical and Molecular Features in Children With Medulloblastoma Treated With Risk-Adapted Therapy: Results of an International Phase III Trial (SJMB03). J. Clin. Oncol. *39*, 822–835. https://doi.org/10.1200/JCO.20.01372.

3. Michalski, J.M., Janss, A.J., Vezina, L.G., Smith, K.S., Billups, C.A., Burger, P.C., Embry, L.M., Cullen, P.L., Hardy, K.K., Pomeroy, S.L., et al. (2021). Children's Oncology Group Phase III Trial of Reduced-Dose and Reduced-Volume Radiotherapy With Chemotherapy for Newly Diagnosed Average-Risk Medulloblastoma. J. Clin. Oncol. *39*, 2685–2697. https://doi.org/10.1200/JCO.20.02730.

4. Robinson, G.W., Rudneva, V.A., Buchhalter, I., Billups, C.A., Waszak, S.M., Smith, K.S., Bowers, D.C., Bendel, A., Fisher, P.G., Partap, S., et al. (2018). Risk-adapted therapy for young children with medulloblastoma (SJYC07): therapeutic and molecular outcomes from a multicentre, phase 2 trial. Lancet Oncol. *19*, 768–784. https://doi.org/10.1016/S1470-2045(18)30204-3.

5. King, A.A., Seidel, K., Di, C., Leisenring, W.M., Perkins, S.M., Krull, K.R., Sklar, C.A., Green, D.M., Armstrong, G.T., Zeltzer, L.K., et al. (2017). Long-term neurologic health and psychosocial function of adult survivors of childhood medulloblastoma/PNET: a report from the Childhood Cancer Survivor Study. Neuro Oncol. *19*, 689–698. https://doi.org/10.1093/neuonc/now242.

6. Leary, S.E.S., Packer, R.J., Li, Y., Billups, C.A., Smith, K.S., Jaju, A., Heier, L., Burger, P., Walsh, K., Han, Y., et al. (2021). Efficacy of Carboplatin and Isotretinoin in Children With High-risk Medulloblastoma: A Randomized Clinical Trial From the Children's Oncology Group. JAMA Oncol. *7*, 1313–1321. https://doi.org/10.1001/jamaoncol.2021.2224.

7. Levy, A.S., Krailo, M., Chi, S., Villaluna, D., Springer, L., Williams-Hughes, C., Fouladi, M., and Gajjar, A. (2021). Temozolomide with irinotecan versus temozolomide, irinotecan plus bevacizumab for recurrent medulloblastoma of childhood: Report of a COG randomized Phase II screening trial. Pediatr. Blood Cancer *68*, e29031. https://doi.org/10.1002/pbc.29031.

8. Maude, S.L., Laetsch, T.W., Buechner, J., Rives, S., Boyer, M., Bittencourt, H., Bader, P., Verneris, M.R., Stefanski, H.E., Myers, G.D., et al. (2018). Tisagenlecleucel in Children and Young Adults with B-Cell Lymphoblastic Leukemia. N. Engl. J. Med. *378*, 439–448. https://doi.org/10.1056/NEJMoa1709866.

9. Del Bufalo, F., De Angelis, B., Caruana, I., Del Baldo, G., De Ioris, M.A., Serra, A., Mastronuzzi, A., Cefalo, M.G., Pagliara, D., Amicucci, M., et al. (2023). GD2-CART01 for Relapsed or Refractory High-Risk Neuroblastoma. N. Engl. J. Med. *388*, 1284–1295. https://doi.org/10.1056/NEJMoa2210859.

10. Majzner, R.G., Ramakrishna, S., Yeom, K.W., Patel, S., Chinnasamy, H., Schultz, L. M., Richards, R.M., Jiang, L., Barsan, V., Mancusi, R., et al. (2022). GD2-CAR T cell therapy for H3K27M-mutated diffuse midline gliomas. Nature *603*, 934–941. https://doi.org/10.1038/s41586-022-04489-4.

11. Vitanza, N.A., Johnson, A.J., Wilson, A.L., Brown, C., Yokoyama, J.K., Künkele, A., Chang, C.A., Rawlings-Rhea, S., Huang, W., Seidel, K., et al. (2021). Locoregional infusion of HER2-specific CAR T cells in children and young adults with recurrent or refractory CNS tumors: an interim analysis. Nat. Med. *27*, 1544–1552. https://doi.org/10.1038/s41591-021-01404-8.

12. Vitanza, N.A., Wilson, A.L., Huang, W., Seidel, K., Brown, C., Gustafson, J.A., Yokoyama, J.K., Johnson, A.J., Baxter, B.A., Koning, R.W., et al. (2023). Intraventricular B7-H3 CAR T Cells for Diffuse Intrinsic Pontine Glioma: Preliminary First-in-Human Bioactivity and Safety. Cancer Discov. *13*, 114–131. https://doi.org/10.1158/2159-8290.CD-22-0750.

13. Ahmed, N., Brawley, V., Hegde, M., Bielamowicz, K., Kalra, M., Landi, D., Robertson, C., Gray, T.L., Diouf, O., Wakefield, A., et al. (2017). HER2-Specific Chimeric Antigen Receptor-Modified Virus-Specific T Cells for Progressive Glioblastoma: A Phase 1 Dose-Escalation Trial. JAMA Oncol. *3*, 1094–1101. https://doi.org/10.1001/jamaoncol.2017.0184.

14. Li, N., Fu, H., Hewitt, S.M., Dimitrov, D.S., and Ho, M. (2017). Therapeutically targeting glypican-2 via single-domain antibody-based chimeric antigen receptors and immunotoxins in neuroblastoma. Proc. Natl. Acad. Sci. USA *114*, E6623–E6631. https://doi.org/10.1073/pnas.1706055114.

15. Bosse, K.R., Raman, P., Zhu, Z., Lane, M., Martinez, D., Heitzeneder, S., Rathi, K.S., Kendsersky, N.M., Randall, M., Donovan, L., et al. (2017). Identification of GPC2 as an Oncoprotein and Candidate Immunotherapeutic Target in High-Risk Neuroblastoma. Cancer Cell *32*, 295–309.e12. https://doi.org/10.1016/j.ccell.2017.08.003.

16. Heitzeneder, S., Bosse, K.R., Zhu, Z., Zhelev, D., Majzner, R.G., Radosevich, M.T., Dhingra, S., Sotillo, E., Buongervino, S., Pascual-Pasto, G., et al. (2022). GPC2-CAR T cells tuned for low antigen density mediate potent activity against neuroblastoma without toxicity. Cancer Cell *40*, 53–69.e9. https://doi.org/10.1016/j.ccell.2021.12.005.

17. Li, N., Torres, M.B., Spetz, M.R., Wang, R., Peng, L., Tian, M., Dower, C.M., Nguyen, R., Sun, M., Tai, C.H., et al. (2021). CAR T cells targeting tumor-associated exons of glypican 2 regress neuroblastoma in mice. Cell Rep. Med. *2*, 100297. https://doi.org/10.1016/j.xcrm.2021.100297.

18. Sun, M., Cao, Y., Okada, R., Reyes-González, J.M., Stack, H.G., Qin, H., Li, N., Seibert, C., Kelly, M.C., Ruppin, E., et al. (2023). Preclinical optimization of a GPC2-targeting CAR T-cell therapy for neuroblastoma. J. Immunother. Cancer *11*, e005881. https://doi.org/10.1136/jitc-2022-005881.

19. Tian, M., Cheuk, A.T., Wei, J.S., Abdelmaksoud, A., Chou, H.C., Milewski, D., Kelly, M.C., Song, Y.K., Dower, C.M., Li, N., et al. (2022). An optimized bicistronic chimeric antigen receptor against GPC2 or CD276 overcomes heterogeneous expression in neuroblastoma. J. Clin. Investig. *132*, e155621. https://doi.org/10.1172/JCI155621.

20. Foster, J.B., Griffin, C., Rokita, J.L., Stern, A., Brimley, C., Rathi, K., Lane, M. V., Buongervino, S.N., Smith, T., Madsen, P.J., et al. (2022). Development of GPC2-directed chimeric antigen receptors using mRNA for pediatric brain tumors. J. Immunother. Cancer *10*, e004450. https://doi.org/10.1136/jitc-2021-004450.

21. Straathof, K., Flutter, B., Wallace, R., Jain, N., Loka, T., Depani, S., Wright, G., Thomas, S., Cheung, G.W.K., Gileadi, T., et al. (2020). Antitumor activity without on-target off-tumor toxicity of GD2-chimeric antigen receptor T cells in patients with neuroblastoma. Sci. Transl. Med. *12*, eabd6169. https://doi.org/10.1126/scitranslmed.abd6169.

22. Majzner, R.G., Theruvath, J.L., Nellan, A., Heitzeneder, S., Cui, Y., Mount, C.W., Rietberg, S.P., Linde, M.H., Xu, P., Rota, C., et al. (2019). CAR T Cells Targeting B7-H3, a Pan-Cancer Antigen, Demonstrate Potent Preclinical Activity Against Pediatric Solid Tumors and Brain Tumors. Clin. Cancer Res. *25*, 2560–2574. https://doi.org/10.1158/1078-0432.CCR-18-0432.

23. Ivanov, D.P., Coyle, B., Walker, D.A., and Grabowska, A.M. (2016). In vitro models of medulloblastoma: Choosing the right tool for the job. J. Biotechnol. *236*, 10–25. https://doi.org/10.1016/j.jbiotec.2016.07.028.

24. Fiaschetti, G., Castelletti, D., Zoller, S., Schramm, A., Schroeder, C., Nagaishi, M., Stearns, D., Mittelbronn, M., Eggert, A., Westermann, F., et al. (2011). Bone morphogenetic protein-7 is a MYC target with prosurvival functions in childhood medulloblastoma. Oncogene *30*, 2823–2835. https://doi.org/10.1038/onc.2011.10.

25. Snuderl, M., Batista, A., Kirkpatrick, N.D., Ruiz de Almodovar, C., Riedemann, L., Walsh, E.C., Anolik, R., Huang, Y., Martin, J.D., Kamoun, W., et al. (2013). Targeting Placental Growth Factor/Neuropilin 1 Pathway Inhibits Growth and Spread of Medulloblastoma. Cell *152*, 1065–1076. https://doi.org/10.1016/j.cell.2013.01.036.

26. Downing, J.R., Wilson, R.K., Zhang, J., Mardis, E.R., Pui, C.H., Ding, L., Ley, T.J., and Evans, W.E. (2012). The Pediatric Cancer Genome Project. Nat. Genet. *44*, 619–622. https://doi.org/10.1038/ng.2287.

27. Powderly, J., Cote, G., Flaherty, K., Szmulewitz, R.Z., Ribas, A., Weber, J., Loo, D., Baughman, J., Chen, F., Moore, P., et al. (2015). Interim results of an ongoing Phase I, dose escalation study of MGA271 (Fc-optimized humanized anti-B7-H3 monoclonal antibody) in patients with refractory B7-H3-expressing neoplasms or neoplasms whose vasculature expresses B7-H3. J. Immunother. Cancer *3*, O8. https://doi.org/10.1186/2051-1426-3-s2-o8.

28. Vitanza, N.A., Wilson, A.L., Huang, W., Seidel, K., Brown, C., Gustafson, J.A., Yokoyama, J.K., Johnson, A.J., Baxter, B.A., Koning, R.W., et al. (2023). Intraventricular B7-H3 CAR T cells for diffuse intrinsic pontine glioma: preliminary first-in-human bioactivity and safety. Cancer Discov. *13*, 114–131. https://doi.org/10.1158/2159-8290.CD-22-0750.

29. Monje, M., Mahdi, J., Majzner, R., Yeom, K.W., Schultz, L.M., Richards, R.M., Barsan, V., Song, K.W., Kamens, J., Baggott, C., et al. (2025). Intravenous and intracranial GD2-CAR T cells for H3K27M+ diffuse midline gliomas. Nature *637*, 708–715. https://doi.org/10.1038/s41586-024-08171-9.

30. Wherry, E.J., Ha, S.J., Kaech, S.M., Haining, W.N., Sarkar, S., Kalia, V., Subramaniam, S., Blattman, J.N., Barber, D.L., and Ahmed, R. (2007). Molecular signature of CD8+ T cell exhaustion during chronic viral infection. Immunity *27*, 670–684. https://doi.org/10.1016/j.immuni.2007.09.006.

31. Li, N., Nguyen, R., Thiele, C.J., and Ho, M. (2021). Preclinical testing of chimeric antigen receptor T cells in neuroblastoma mouse models. STAR Protoc. *2*, 100942. https://doi.org/10.1016/j.xpro.2021.100942.

32. Brehm, M.A., Kenney, L.L., Wiles, M.V., Low, B.E., Tisch, R.M., Burzenski, L., Mueller, C., Greiner, D.L., and Shultz, L.D. (2019). Lack of acute xenogeneic graft- versus-host disease, but retention of T-cell function following engraftment of human peripheral blood mononuclear cells in NSG mice deficient in MHC class I and II expression. FASEB J. *33*, 3137–3151. https://doi.org/10.1096/fj.201800636R.

33. DeVos, S.L., and Miller, T.M. (2013). Direct intraventricular delivery of drugs to the rodent central nervous system. J. Vis. Exp. *75*, e50326. https://doi.org/10.3791/50326.

34. Stuart, T., Butler, A., Hoffman, P., Hafemeister, C., Papalexi, E., Mauck, W.M., 3rd, Hao, Y., Stoeckius, M., Smibert, P., and Satija, R. (2019). Comprehensive Integration of Single-Cell Data. Cell *177*, 1888–1902.e21. https://doi.org/10.1016/j.cell.2019.05.031.

35. Young, M.D., and Behjati, S. (2020). SoupX removes ambient RNA contamination from droplet-based single-cell RNA sequencing data. GigaScience *9*, giaa151. https://doi.org/10.1093/gigascience/giaa151.

36. Wolock, S.L., Lopez, R., and Klein, A.M. (2019). Scrublet: Computational Identification of Cell Doublets in Single-Cell Transcriptomic Data. Cell Syst. *8*, 281–291.e9. https://doi.org/10.1016/j.cels.2018.11.005.

37. Hao, Y., Hao, S., Andersen-Nissen, E., Mauck, W.M., Lee, M.J., Wilk, A.J., Darby, C., Zager, M., , et al.Zheng, S., Butler, A. (2021). Integrated analysis of multimodal single-cell data. Cell *184*, 3573–3587.e29. https://doi.org/10.1016/j.cell.2021.04.048.

OMTON, Volume *33*

Supplemental information

GPC2-CAR T cells have potent preclinical activity against orthotopic medulloblastoma xenografts

Reona Okada, Mia Fanuzzi, Constanza Rodriguez, Jangsuk Oh, Adhithi Sreenivasan, Hannah G. Stack, Mariela Puebla, Ira Phadke, Allison P. Cole, Shanshan Bradford, Michael C. Kelly, Haiyan Lei, Mitchell Ho, Jennifer A. Cotter, Carol J. Thiele, Xiyuan Zhang, Anandani Nellan, and Rosa Nguyen

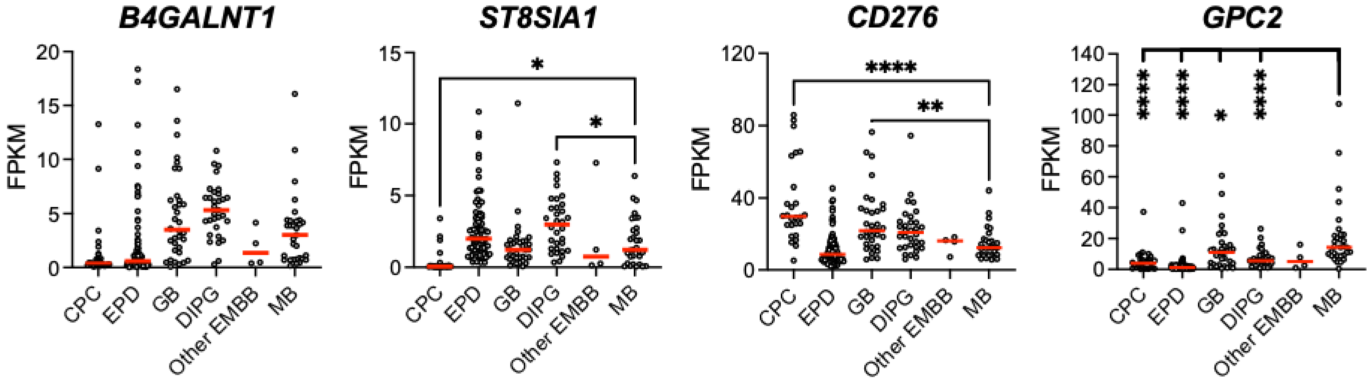

Figure S1: Expression of *B4GLANT1* and *ST8SIA1* (enzymes responsible for GD2 synthesis), *CD276*, and *GPC2* in various primary tumors and derived xenografts and cell lines from patients with choroid plexus carcinoma (CPC), ependymoma (EPD), glioblastoma (GB), diffuse intrinsic pontine glioma (DIPG), other embryonal brain tumors (EMBB), and medulloblastoma (MB) from primary patient samples obtained through the St. Jude Pediatric Cancer Knowledge Base portal.

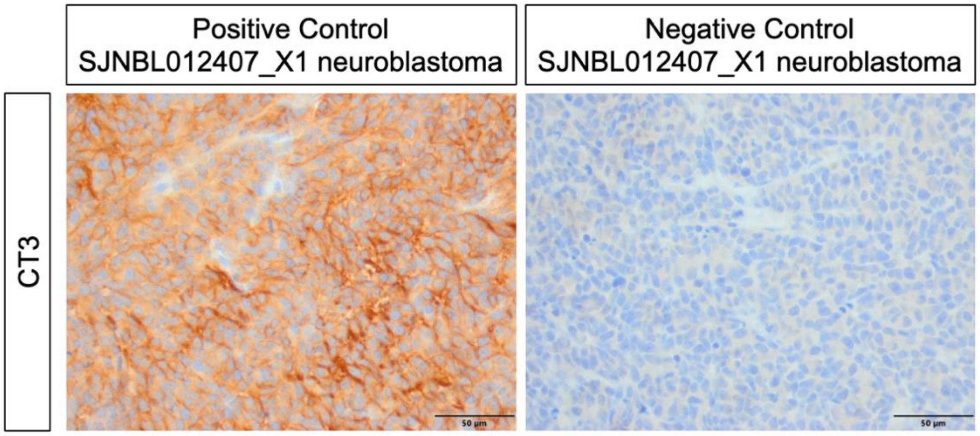

Figure S2: The positive control tissue consisted of SJNBL012407_X1 neuroblastoma cells, which demonstrated diffuse and strong CT3 positivity with a cytoplasmic to membranous staining pattern (CT3, 1:1000, BioX BE0402, mouse IgG1, using the M.O.M. ImmPRESS Kit and Bond Polymer Refine Detection Kit). The negative control was performed on the same tissue by substituting the primary antibody with a nonclonal, isotype-matched mouse antibody.

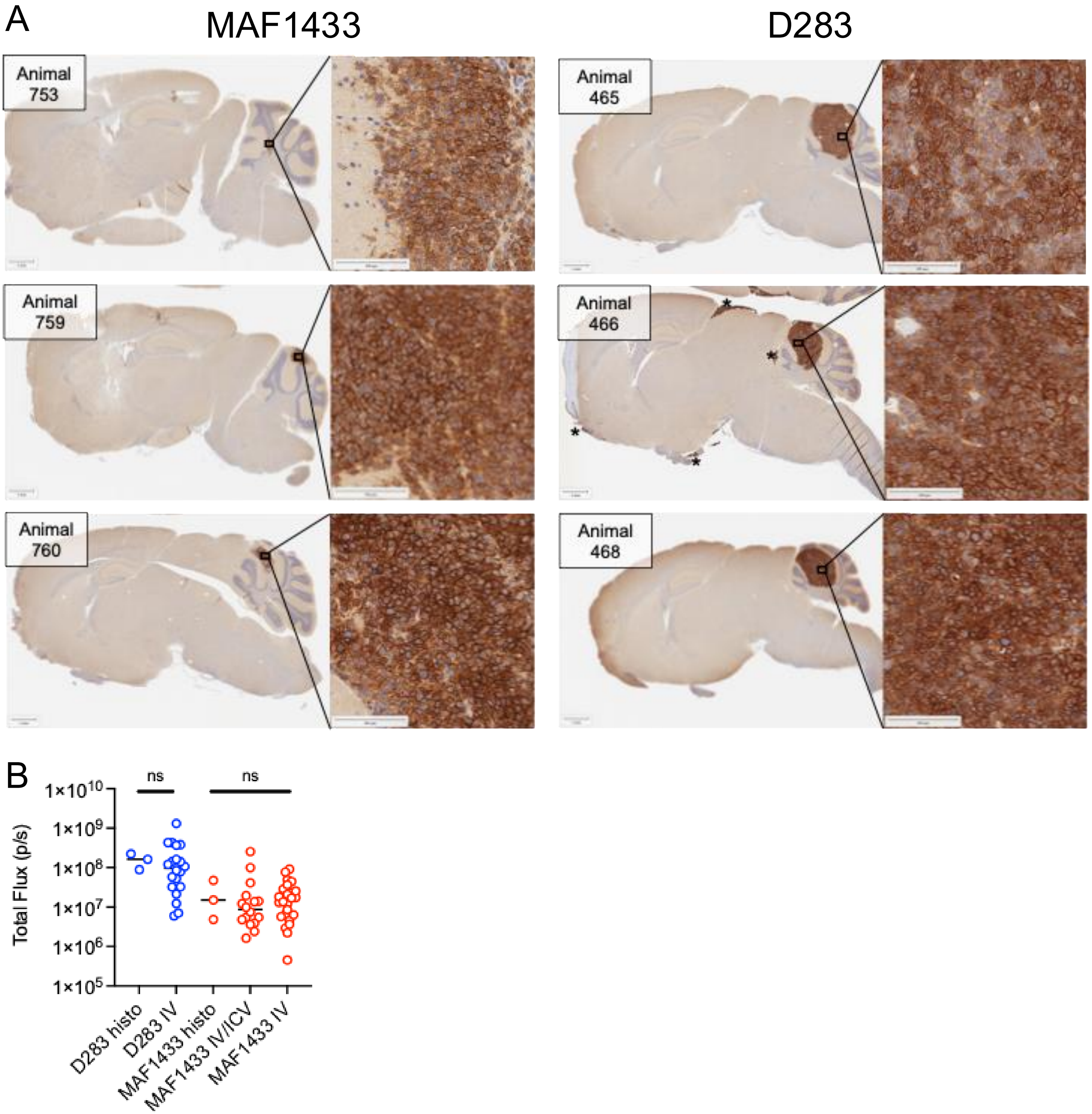

Figure S3: (A) *In vivo* GPC2 expression in MAF1433 and D283. Mice were injected with MAF1433 and D283 cells, and tumors were harvested at the time point corresponding to when CAR T-cell treatment would have been administered. Immunohistochemistry performed on these tissues revealed homogeneous and strong GPC2 expression in both models. **(B)** Animals shown in (A) were also subjected to IVIS bioluminescence imaging (BLI). The average BLI signal was compared to the baseline signal of mice treated in this study. No significant difference in average signal was observed, indicating comparable tumor burden across studies.

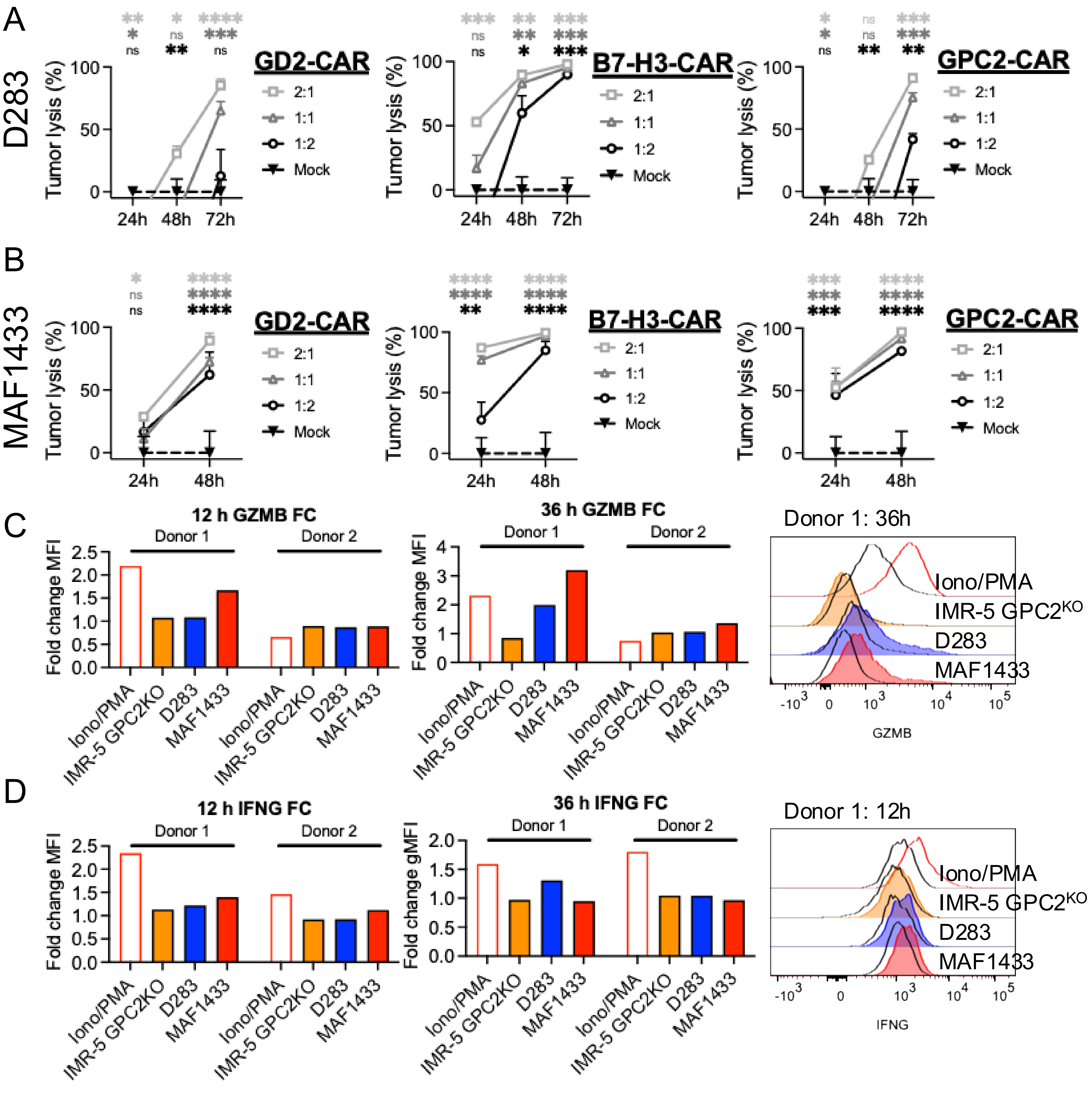

Figure S4: *In vitro* CAR T-cell killing against **(A)** D283 and **(B)** MAF1433 (second replicate with independent donor). Intracellular **(C)** granzyme B and **(D)** interferon gamma levels of GPC2-CAR T-cells compared to mock control T-cells are shown as fold change at 12 and 36 hours post-co-culture. A representative flow plot is shown for each cytokine. The black curve is the isotype control for each condition.

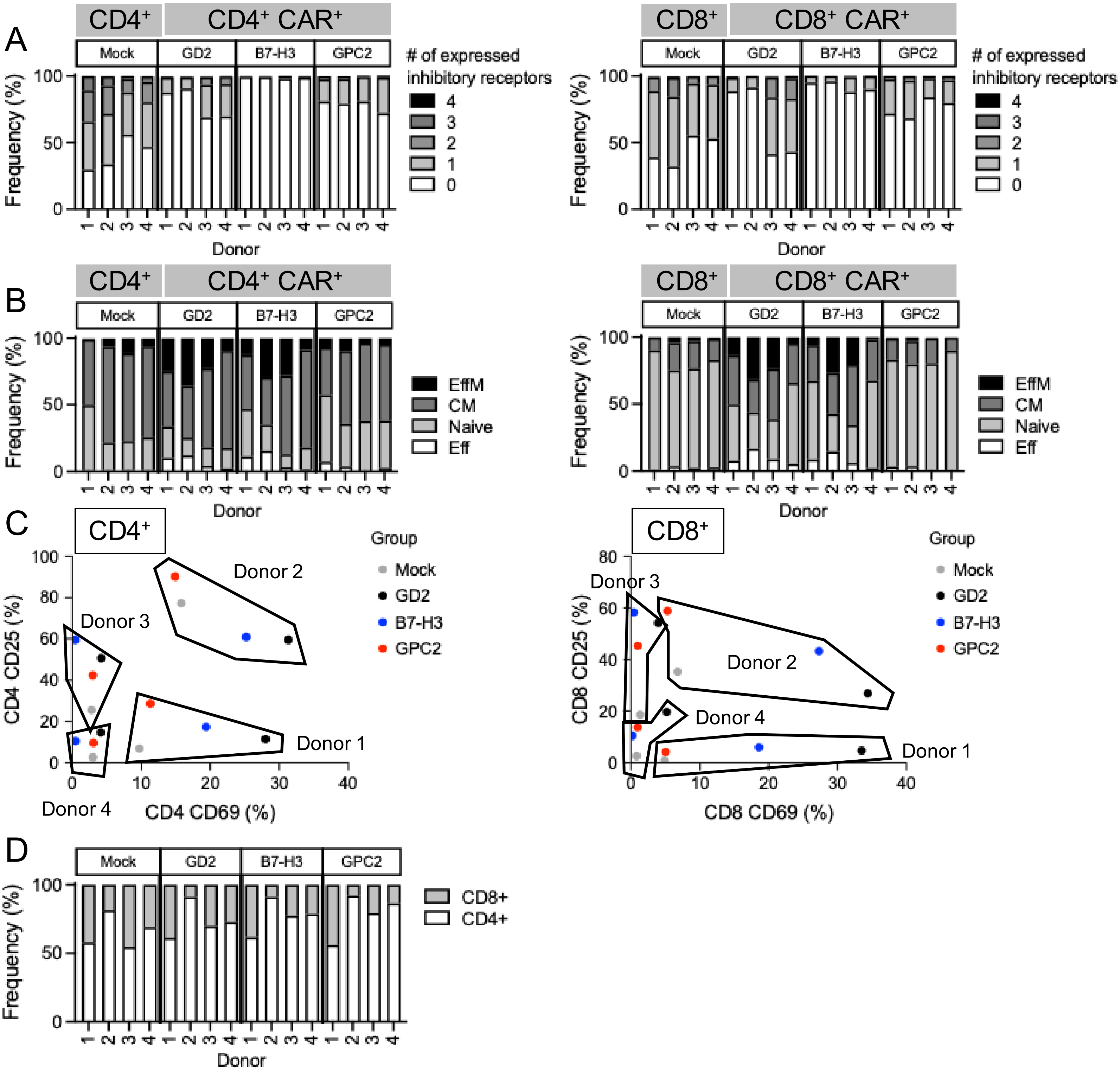

Figure S5: Phenotype analysis of different T-cell products after production. **(A)** The fraction of cells with single, double, triple, or quadruple positivity for LAG3, TIM3, CD39, and PD-1 is plotted for four independent donors and shown for CD4 and CD8 T-cells. **(B)** Fractions of $CD62L^-$ $CD45RA^-$ effector memory (EffM), $CD62L^+$ $CD45RA^-$ central memory (CM), $CD62L^+$ $CD45RA^+$ naïve, and $CD62L^-$ $CD45RA^+$ effector T-cells (Eff) across the four donors from (A). **(C)** CD25 and CD69 positivity across CARs and donors is shown for CD4 and CD8 T-cells separately. **(D)** Frequency of CD8- and CD4-positive T-cells by CAR group.

A D283 rechallenge assay

Tukey's multiple comparisons test	Mean Diff.	95.00% CI of diff.	Summary	Adj. P Value
Mock vs. B7-H3-CAR	34.17	28.15 to 40.20	****	<0.0001
Mock vs. GD2-CAR	28.36	22.91 to 33.81	****	<0.0001
Mock vs. GPC2-CAR	-11.36	-13.79 to -8.929	****	<0.0001
B7-H3-CAR vs. GD2-CAR	-7.330	-8.056 to -6.604	****	<0.0001
B7-H3-CAR vs. GPC2-CAR	-45.53	-53.89 to -37.18	****	<0.0001
GD2-CAR vs. GPC2-CAR	-39.72	-47.45 to -31.99	****	<0.0001

B MAF1433 rechallenge assay

Tukey's multiple comparisons test	Mean Diff.	95.00% CI of diff.	Summary	Adj. P Value
Mock vs. B7H3 CAR	4.714	3.991 to 5.438	****	<0.0001
Mock vs. GD2 CAR	4.572	3.773 to 5.370	****	<0.0001
Mock vs. GPC2 CAR	2.797	2.343 to 3.250	****	<0.0001
B7H3 CAR vs. GD2 CAR	-0.1426	-0.2402 to -0.04505	**	0.0014
B7H3 CAR vs. GPC2 CAR	-1.917	-2.195 to -1.640	****	<0.0001
GD2 CAR vs. GPC2 CAR	-1.775	-2.129 to -1.420	****	<0.0001

C

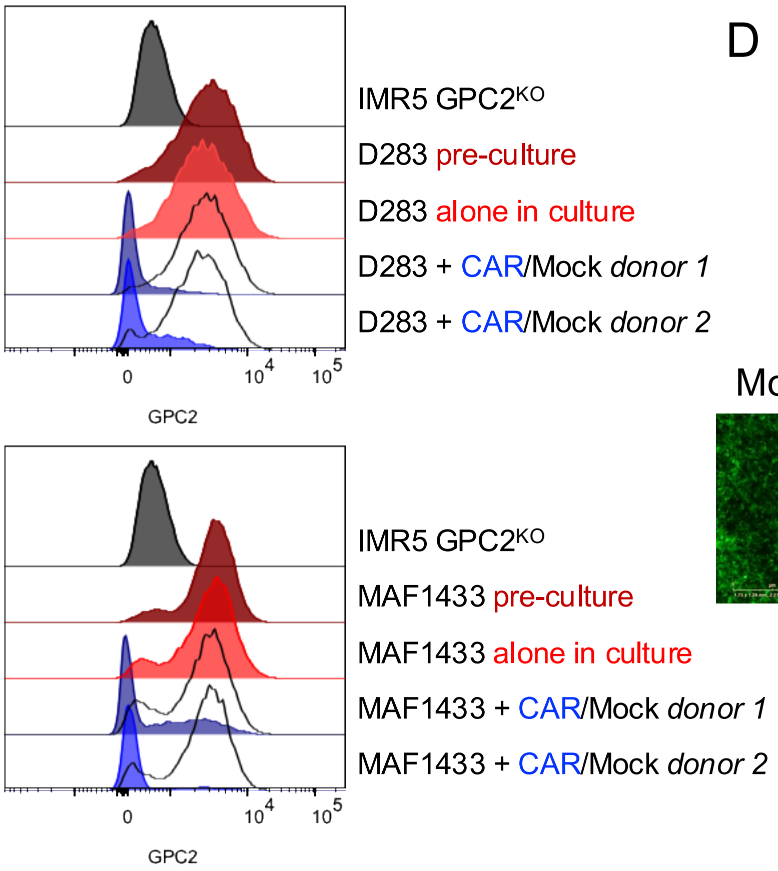

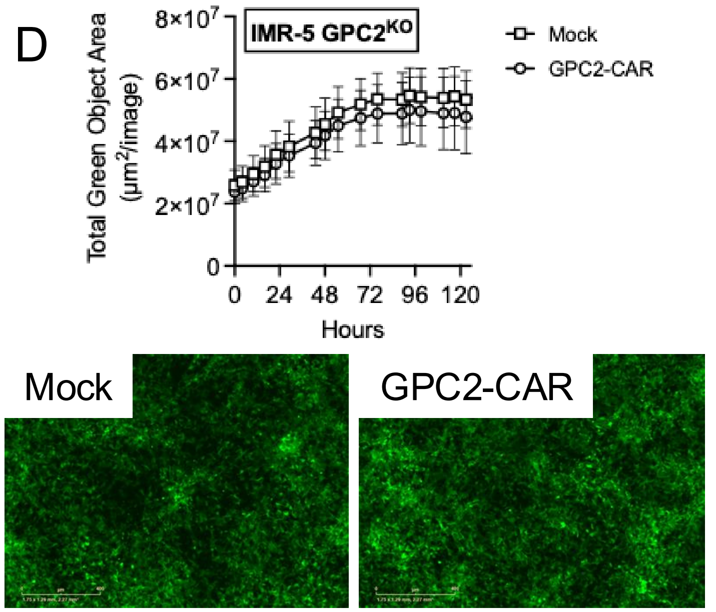

Figure S6: Tukey's multiple comparisons test of each treatment group for the **(A)** D283 and **(B)** MAF1433 tumor rechallenge assay. These statistics correspond to results shown in main Fig. 2E-F. **(C)** GPC2 expression in surviving D283 and MAF1433 cell clones after co-culture with Mock or GPC2-CAR T-cells. These cells downregulate GPC2 under CAR-mediated selection pressure. **(D)** GPC2-CAR T-cell killing against the GPC2 knockout line IMR-5 $GPC2^{KO}$. IMR-5 $GPC2^{KO}$ cells are resistant to GPC2-CAR cytotoxicity. Using Incucyte live-cell imaging, tumor growth curves are similar for mock T-cell-treated or GPC2-CAR-treated conditions. Representative fluorescence images of a co-culture well are shown at the bottom panel, where green signals represent tumor cells.

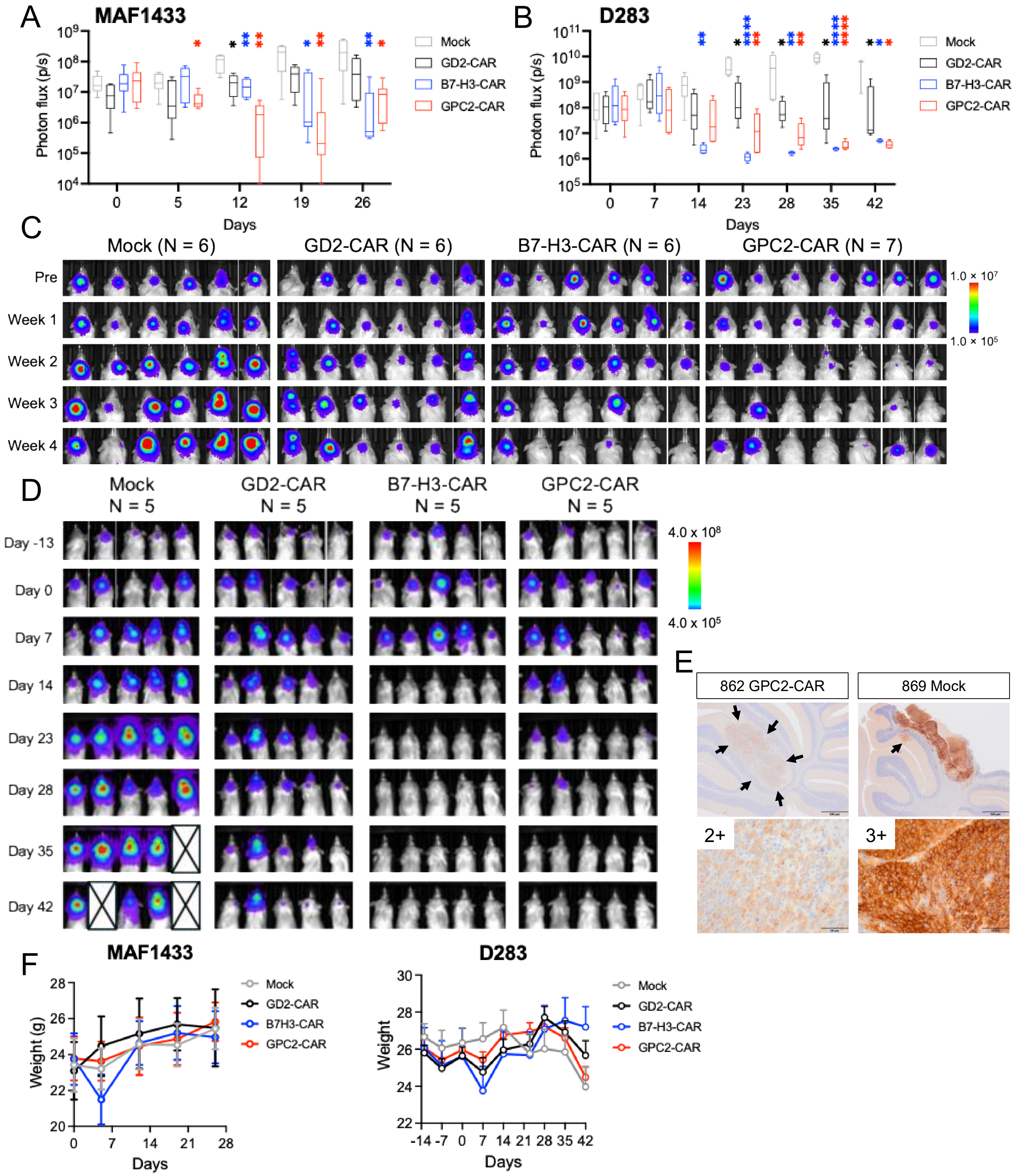

Figure S7: Bar graph plot of the BLI signal of **(A)** MAF1433 and **(B)** D283 tumors. Two-way ANOVA with multiple comparisons; * $P < 0.05$; ** $P < 0.01$; **** $P < 0.0001$. Corresponding IVIS bioluminescence images of **(C)** MAF1433 and **(D)** D283-bearing mice during CAR T-cell treatment. **(E)** Histological review of MAF1433 mice treated with GPC2-CAR and IVIS signals above background demonstrates tumor cells in the posterior fossa with marked downregulation of GPC2 compared to mock-treated animals. **(F)** Longitudinal body weight measurements of study mice.

A

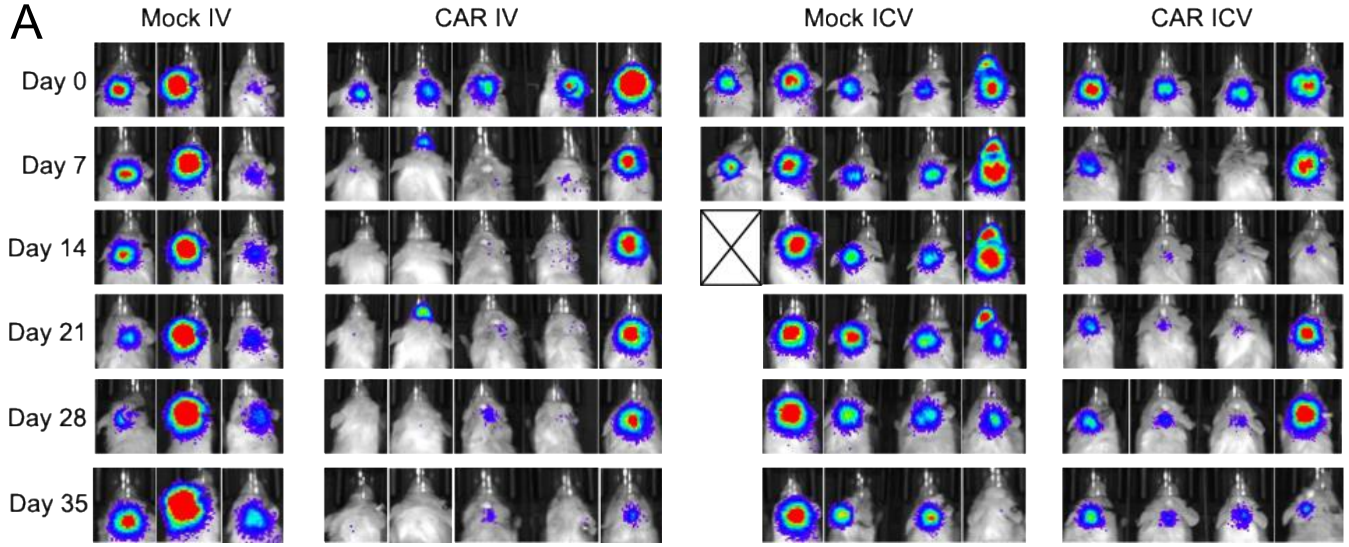

B

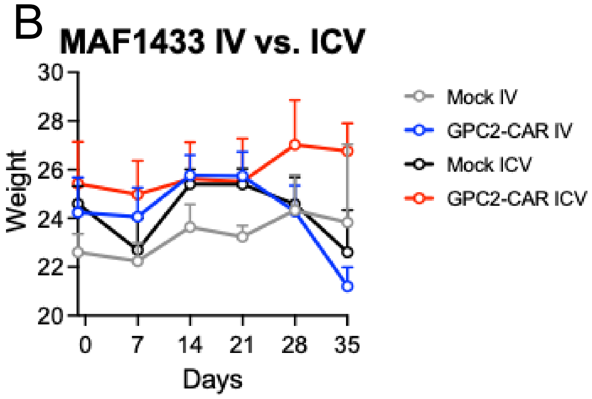

Figure S8: (A) Corresponding IVIS bioluminescence images of orthotopic MAF1433-bearing mice treated with mock or GPC2-CAR T-cells via intravenous (IV) or intraventricular (ICV) injection. **(B)** Longitudinal bodyweight measurement of study mice.

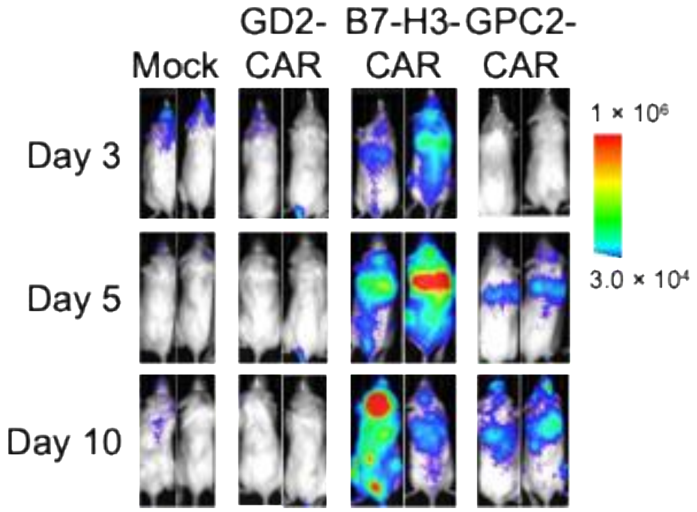

Figure S9: CAR T-cell tracking in the orthotopic D283 wildtype model. The IVIS BLI signal indicates T-cells. Over time, T-cells home to the tumor and lymphoid organs to expand.

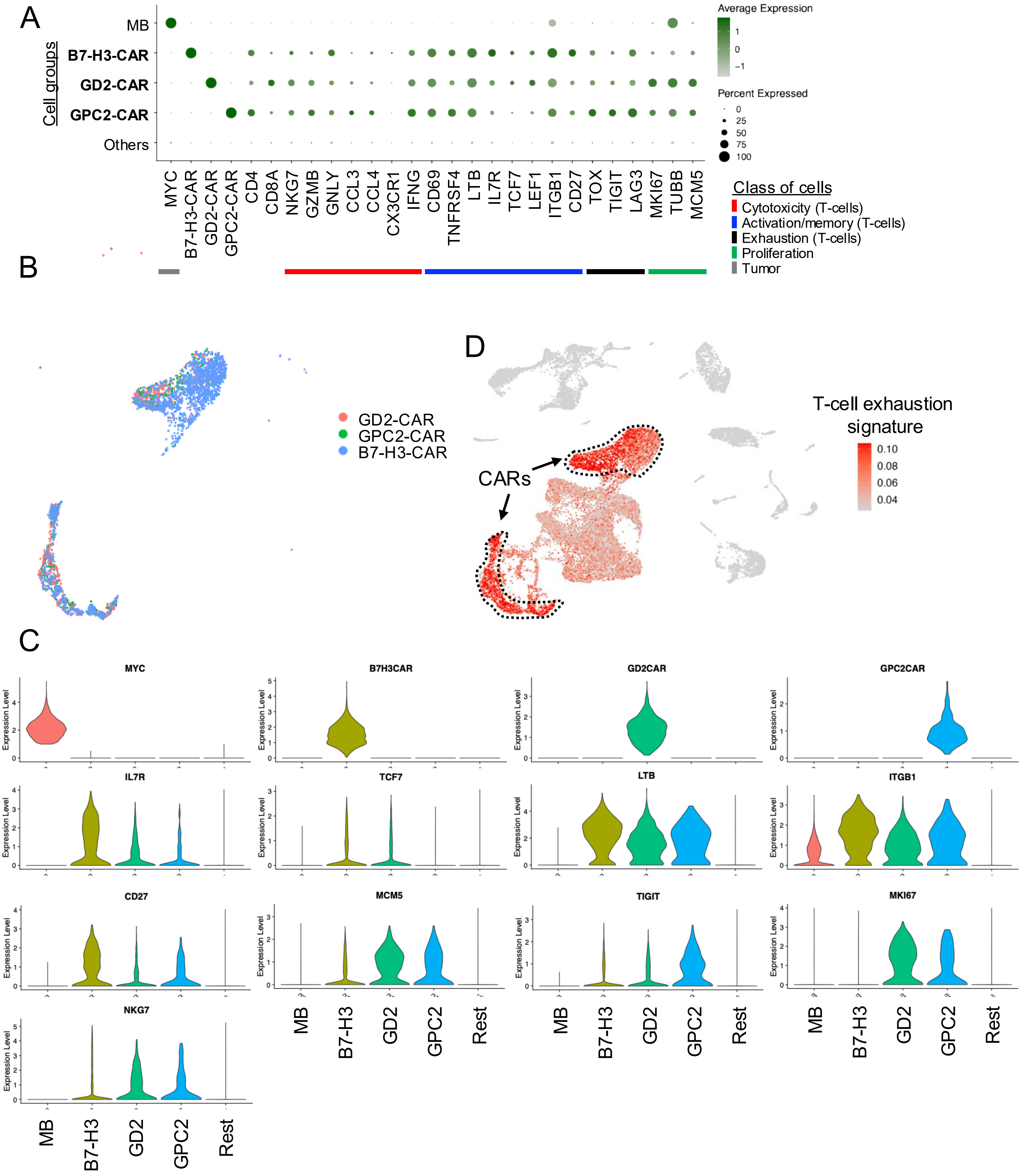

Figure S10: (A) The dot plot exhibits the relative expression of key genes organized by cellular processes. The dot size indicates the percentage of cells expressing each gene, and the dot color represents the average expression levels. **(B)** Integrated and reclustered CAR populations. **(C)** Violin plot of several key genes. **(D)** Expression levels of T-cell exhaustion gene signature in the single-cell data set.

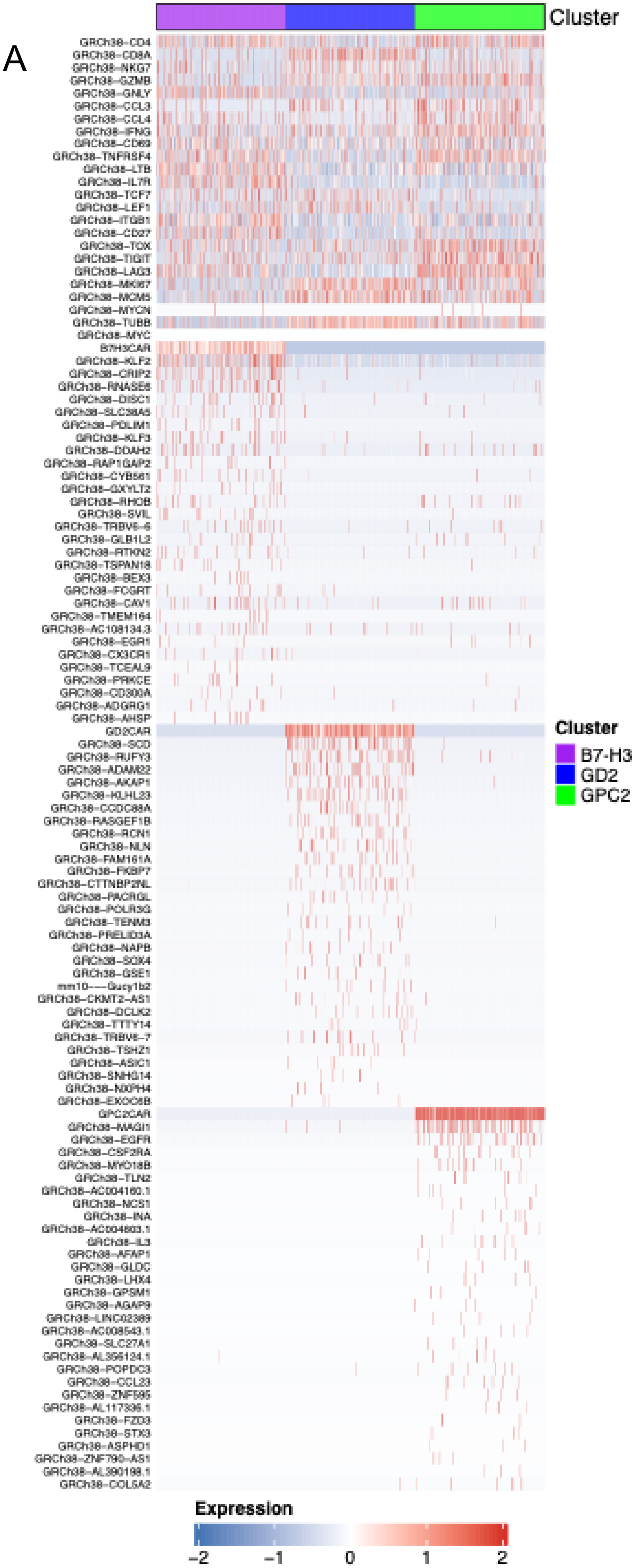

Figure S11: Heat map of top 30 genes for each CAR cluster, including a selection of genes relevant for T-cell function.

Table S1: Methylation profiles of D283 and MAF1433 categorize both lines as medulloblastoma non-WNT/non-SHH activated group 3 tumors.

	Heidelberg Epigenomics CNS Tumor Classifier	NIH Bethesda CNS Tumor Classifier
D283		
Medulloblastoma	0.906067	0.93
Non-WNT/nonSHH activated	0.825379	
Group 3	0.777417	
Subclass 2	0.662449	
MAF1433		
Medulloblastoma	0.830693	0.95
Non-WNT/nonSHH activated	0.746062	
Group 3	0.696090	
Subclass 2	0.564192	

Table S2: Pathological review of mouse cerebella post GPC2-CAR T-cell treatment.

Animal #	Treatment group	H&E, cerebellum
763	Mock IV	Tumor (2x1mm)
766	Mock IV	Tumor (2x1mm)
769	Mock IV	Tumor (1x1mm)
751	CAR IV	No tumor
752	CAR IV	No tumor
764	CAR IV	No tumor
765	CAR IV	No tumor
773	CAR IV	No tumor
754	Mock ICV	No tumor
758	Mock ICV	Tumor (2x2mm)
771	Mock ICV	Tumor (2x2mm)
772	Mock ICV	Tumor (2x2mm)
774	Mock ICV	No tumor
755	CAR ICV	Tumor (2x1mm)
756	CAR ICV	Tumor (1x1mm)
757	CAR ICV	Tumor (2x1mm)
761	CAR ICV	Tumor (1x1mm)
762	CAR ICV	Tumor (2x1mm)

IV: intravenous; ICV: intracerebroventricular

Table S3: Cluster annotation of single-cell RNA-seq data set, including cluster-defining genes.

Cluster	Species	Annotation	Differentially expressed genes
0	human	MB	*SOX4*
1	human	MB	*TTR*
2	human	B7-H3-CAR	*B7-H3-CAR, GNLY, CCL5, CD4, CD8*
3	human	MB	*SOX4, MYC, ODC1*
4	murine	M2 TAMs	*Mrc1, Apoe, Ccl8, CD68, Fcgr2b, Ccl2, Ccl7*
5	human	MB	*MYC*
6	murine	Astrocytes	*Dbi, Nnat, Rsph1, Mt3, Tmem212*
7_0	human	GD2-CAR, proliferating	*GD2-CAR, TOP2A, CCL4, CD4, CD8*
7_1	human	MB, all CARs, proliferating	*MYC, B7-H3-CAR, GD2-CAR, GPC2-CAR, CD3D*
7_2	human	GD2-CAR, B7-H3-CAR, proliferating	*B7-H3-CAR, GD2-CARCD3D*
7_3	human	B7-H3-CAR, proliferating	*B7-H3-CAR, TOP2A*
7_4	human	MB, proliferating	*MYC, TOP2A*
7_5	human	GD2-CAR, proliferating	*GD2-CAR, TOP2A*
7_6	human	GD2-CAR, proliferating	*GD2-CAR, TOP2A*
8	murine	Endothelial cells	*Bsg, Ly6c1, Itm2a, Cldn5, Flt1*
9	murine	CNS-associated macrophages	*Lyz2, Cd74, H2-Aa, H2-Ab1, H2-Eb1*
10	murine	Oligodendrocytes	*Plp1, Cryab, Aplp1, Tubb4a, Car2*
11	murine	Microglia	*Hexb, P2ry12, Cst3, Cx3cr1, Csmd3*
12	human	All CARs	*B7-H3-CAR, GD2-CAR, GPC2-CAR, CD3D, XCL1*
13	human	MB	*MLLT11*
14_0	human	B7-H3-CAR	*B7-H3-CAR, CD3D*
14_1	human	MB	*MYC*
14_2	human	MB, proliferating	*MYC, TOP2A*
14_3	human	MB	*MYC*
14_4	human	MB	*MYC*
15_0	human	GD2-CAR, proliferating	*GD2-CAR, HMGB2, TOP2A, MKI67*
15_1	human	GD2-CAR, proliferating	*GD2-CAR, HMGB2, TOP2A, MKI67*
15_2	human	B7-H3-CAR, proliferating	*B7-H3-CAR, TOP2A*
15_3	human	MB	*MYC*
15_4	human	MB, proliferating	*MYC, TOP2A*
15_5	human	MB, proliferating	*MYC, TOP2A*
16	murine	Astrocytes	*Lsamp, Atp1a2, Grid2, Atp1b2, Hsd11b1*
17	murine	PMN	*S100a9, S100a8, Retnlg, Ngp, Il1b, Lcn2, Cxcl2*
18	human	MB	*TOP2A, HMGB2, MKI67*
19	murine	Monocytes	*Plac8, S100a4, Gpx1, Lgals3, Cebpb*
20	murine	Astrocytes	*Scl1a3, Gpr37l1, Aldoc, Timp4, Plpp3*
21	murine	Astrocytes	*Dbi, Mt3, Nnat*
22	murine	Pericytes	*Vtn, Rgs5, Myl9, Acta2, Cald1*
23	murine	Antigen-presenting cells	*H2-Aa, Cd74, H2-Ab1, H2-Eb1, Ifi30, Lsp1*
24	murine	Fibroblasts	*Mgp, Igfbp6, Rbp1*
25	murine	Neurons	*Atp5g1, Ppp1r1b, Fabp3, Mdh1*
26	murine	Pericytes and endothelial cells	*Vtn, Rgs5, Igfbp7, Bsg, Ly6c1, Itm2a, Flt1, Cldn5*
27	murine	Oligodendrocytes	*Ptgds, Apod, Ptn*
28	murine	Oligodendrocytes	*Nkain2, Pcdh9, Lrp1b, Plcl1, Tmeff2*
29	murine	CNS-associated macrophages	*Bst2, Plac8, Irf8, Ly6a, Siglech*
